# The Association Between Parent-to-Child Fear Learning Pathways and Anxiety Sensitivity: A Systematic Review and Meta-analysis

**DOI:** 10.1007/s10567-025-00517-7

**Published:** 2025-04-21

**Authors:** Ena Alcan, Jana Gessner, Giulia Stangier, Christoph Benke, Jonas Busin, Hanna Christiansen, Christiane A. Melzig

**Affiliations:** 1 Department of Clinical Psychology, Experimental Psychopathology, and Psychotherapy, Institute of Psychology, Philipps-University Marburg, Gutenbergstr. 29a, 35037 Marburg, Germany; 2 Department of Biological and Clinical Psychology, Trier University, Trier, Germany; 3 Department of Clinical Child and Adolescent Psychology, Institute of Psychology, Philipps-University Marburg, Marburg, Germany

**Keywords:** Fear, Anxiety sensitivity, Learning pathway, Systematic review, Meta-analysis

## Abstract

Although anxiety sensitivity (AS), or the fear of anxiety-related symptoms, has been identified as a risk factor for the development of anxiety psychopathology, the pathways through which this fear is learned have not been fully elucidated. In the current review and meta-analysis, we aimed to systematically examine the association between parent-to-child fear learning pathways (vicarious learning, negative information, reinforcement, and punishment) and AS. A comprehensive search of literature was conducted in PsychINFO, PubMed, Embase, and Web of Science databases, using search terms combining categories related to fear learning pathways, anxiety-related symptoms, parents, children, and adolescents. Based on this search strategy, 28 studies were identified as relevant, of which 11 were included in the systematic review and 10 in the meta-analysis. The overall findings indicated that parent-to-child fear learning pathways are significantly associated with AS. The meta-analysis demonstrated a small but significant association between fear learning pathways and AS, although the type of fear learning pathway did not significantly moderate this relationship. However, age emerged as a significant moderator, suggesting a stronger association in children and adolescents compared to adults. Given that these findings are primarily based on cross-sectional studies, this review underscores the need for longitudinal and experimental research to further clarify the role of parent-to-child fear learning pathways in anxiety sensitivity. Additionally, a better understanding of these pathways may help inform existing interventions and fear prevention strategies, such as those aimed at reducing parental modeling of fearful behaviors or promoting positive verbal messages about anxiety symptoms.

Everyone at some point might experience bodily symptoms such as heart palpitations, nausea, or abdominal pain. These commonly occurring symptoms can arise for a number of reasons, for example, due to changes in everyday routines, minor ailments, or in response to stressful life events (Asmundson & Taylor, 2005). However, the perception of these bodily symptoms varies greatly among individuals. While some individuals may perceive these symptoms as transient and benign, others may (mis)interpret such harmless bodily symptoms as threatening and indicative of a potentially serious physical harm (e.g., a serious illness, heart attack) or an upcoming panic attack. One trait-like, cognitive variable that might explain the individual difference in tendency to misinterpret and associate benign bodily symptoms with threat is known as anxiety sensitivity (AS) or the fear of anxiety-related symptoms (Reiss et al., 1986). This construct is distinct from other anxiety-related constructs, such as trait anxiety, which refers to a more general tendency to fear a wide range of stressors and across a variety of situations (McNally, 2002; Chorpita et al., 1996; Olatunji & Wolitzky-Taylor, 2009; Weems et al., 1997). In fact, AS is considered to be a dimensional construct, encompassing multiple dimensions of fear, including fear arising from belief that anxiety-related symptoms have harmful physical, social, and cognitive consequences (Rifkin et al., 2015; Taylor et al., 2007; Wheaton et al., 2012). To illustrate, heightened AS is characterized by an excessive fear in response to anxiety-related symptoms (e.g., racing heart, shortness of breath) due to the belief that these symptoms have harmful consequences (e.g., heart attack), whereas low AS lacks this distinctive characterization (Reiss, 1991).

AS is a particularly relevant construct considering that awareness of bodily symptoms and their association with emotions, such as fear and surprise, begin early in childhood. By around age 6, children develop an increasing ability to recognize and interpret bodily sensations (Hietanen et al., 2016; Muris et al., 2000a, 2004; Ollendick et al., 1996). Although some studies have questioned whether younger children can fully grasp the implications of bodily symptoms (Chorpita & Lilienfeld, 1999), evidence suggests that by age 7, children begin associating bodily symptoms with fear, interpreting sensations such as trembling hands, a fast heartbeat, and difficulty breathing as potentially threatening (Mattis & Ollendick, 1997; Muris et al., 2008, 2010b, 2000b; Weems et al., 1998, 2021). Notably, several studies have demonstrated that children and adolescents with heightened AS are at increased risk for developing anxiety-related psychopathology, including panic disorder, generalized anxiety disorder, social anxiety disorder, and separation anxiety (Joiner et al., 2002; Knapp et al., 2016; Kramer & Francis, 2025; Reiss et al., 2001; Weems et al., 1997, 2000; Wolitzky-Taylor et al., 2015). For example, one longitudinal study has shown that heightened AS at the age of 8 years was significantly associated with anxiety symptoms, especially somatic/panic symptoms, separation and generalized anxiety, and social phobia at the age of 10 years (Waszczuk et al., 2013). Similar results have been reported in adolescents, where heightened AS predicted the occurrence and worsening of anxiety symptoms and panic attacks over time, even after controlling for other variables, such as trait anxiety, negative affectivity, depression, and baseline anxiety (Allan et al., 2014; Deacon et al., 2002; Ginsburg & Drake, 2002; Hayward et al., 2000; Lau et al., 1996; Qi et al., 2019; Schmidt et al., 2010; van Widenfelt et al., 2002; Weems et al., 2002). Additionally, longitudinal research in non-Western samples has found similar results, showing that AS was predictive of both anxiety and depression symptoms 1 year later among Chinese adolescents in Hong Kong (Ho et al., 2018). Collectively, these studies suggest that elevated AS places children and adolescents at heightened risk for the development of anxiety-related psychopathology (Marin et al., 2008; Qi et al., 2019; Weems et al., 2010).

Considering that AS onsets early and predicts the development of anxiety symptoms and disorders (Ginsburg & Drake, 2002; Knapp et al., 2016; Marin et al., 2008; McLaughlin & Hatzenbuehler, 2009; Noël & Francis, 2011; Qi et al., 2019), it is crucial to understand its etiology. Theoretical models of AS have identified both genetic and environmental influences, with twin studies showing that while genetic factors contribute to AS, environmental influences account for a substantial proportion of the variance, either directly or through interactions with genetic predispositions (Eley et al., 2007; Hettema et al., 2020; Stein et al., 1999; Taylor et al., 2008; Zavos et al., 2012). Given the substantial role of environmental influences, extant research has increasingly focused on the family environment, where studies have consistently found that children of parents with heightened AS tend to have elevated AS levels themselves (Coppola et al., 2018; East et al., 2006; Francis & Noël, 2010; Graham & Weems, 2014; Ollendick & Horsch, 2007; Drake & Kearney, 2008; Tsao et al., 2005). Beyond the association between parent and child AS, a few studies have explored the role of specific parenting behaviors in relation to AS, reporting significant associations between childhood AS and negative parenting behaviors, such as threatening, hostile, rejecting, controlling behaviors, and corporal punishment (Erozkan, 2012; Gardner & Epkins, 2012; Graham & Weems, 2014; Gray et al., 2010; Nebbitt & Lambert, 2009; Scher & Stein, 2003). While these findings suggest that various parental behaviors may shape the environment that fosters AS, less is known about the specific ways in which children can learn to fear from their parents in the context of AS. Beyond these parenting behaviors, parents’ own fearful responses to anxiety symptoms could also play a role in shaping children’s AS. Thus, it is important to examine whether parental fearful behaviors are linked to children’s and adolescents’ fear of anxiety-related symptoms, and which fear learning pathways may be involved.

Theories of social learning and fear acquisition suggest that children can develop fears not only through direct aversive experiences but also through indirect learning experiences occurring within the family environment (e.g., by observing fearful parental behaviors) (Bandura & Walters, 1977; Rachman, 1977). Drawing on these theories, extensive research has demonstrated that children can learn to fear from their parents through three fear learning pathways: vicarious fear, negative information, and reinforcement or punishment (for a review, see Fisak & Grills-Taquechel, 2007; Ollendick & Muris, 2015; Ollendick & King, 1991; Muris & Field, 2010; Lebowitz et al., 2016; Aktar et al., 2017; Aktar & Pérez-Edgar, 2024). Through vicarious fear pathway, fears can be learned by observing fearful behaviors or fearful reactions in others. For example, research has shown that children who observe their parents displaying fearful facial expressions in response to various stimuli or situations, such as ambiguous toys, unfamiliar animals, or other potentially threatening stimuli (e.g., snakes, spiders, or strangers), subsequently exhibit heightened fear responses to those same stimuli (Askew & Field, 2007; De Rosnay et al., 2006; Dunne & Askew, 2013; Gerull & Rapee, 2002; Marin et al., 2020; Murray et al., 2008). The second fear learning pathway involves learning fear through negative information, where children learn about potential dangers via verbal instructions or fear-relevant information from others (Muris & Field, 2010; for a review, see Percy et al., 2016). Research has shown that children who receive negative information from their parents about the threat posed by an unfamiliar stimulus, such as a novel toy or animal, are more likely to develop fear toward that stimulus (Field & Lawson, 2003; Field et al., 2001; Muris & Field, 2010; Muris et al., 2010a). The third fear learning pathway involves parental reinforcement and punishment, wherein parents’ responses shape children’s fear-related behaviors. Parents may inadvertently reinforce fear by providing rewards or special attention when a child expresses fear (positive reinforcement) or by facilitating avoidance of fear-provoking situations, such as allowing the child to stay home from school (negative reinforcement). Conversely, punishment or discouragement of fear expressions, such as criticism or disapproval, may heighten fear by teaching children that expressing fear is socially unacceptable or embarrassing (for a review, see Fisak & Grills-Taquechel, 2007; Öst & Hugdahl, 1981; Kirkby et al., 1995; King et al., 1998; Bilsky et al., 2018a).

Contemporary fear acquisition models suggest that these learning pathways operate through associative learning mechanisms that allow children to associate parents’ fear behaviors (e.g., observing fearful facial expressions or hearing negative information) with potentially threatening stimuli or situations (e.g., ambiguous toys) (Aktar & Pérez-Edgar, 2024; Askew & Field, 2008; Field, 2006; Mineka & Cook, 1993; Mineka & Zinbarg, 2006). Although these models highlight the importance of learning pathways in the learning of fear, relatively fewer studies have examined how these pathways relate to AS. Several individual studies have suggested that vicarious learning, negative information, and reinforcement or punishment pathways may be linked to heightened AS (Ehlers, 1993; Watt et al., 1998), but the existing studies have varied in their methodologies and sample characteristics, making it difficult to draw conclusions about the overall strength and nature of these associations. Thus, a systematic review and meta-analysis would not only consolidate and critically evaluate the existing literature, but also provide a quantitative evaluation of the effects across those studies, offering a more precise estimate of the overall strength of the relationship between parent-to-child fear learning pathways and AS. Additionally, by systematically evaluating the literature, we would get a more comprehensive analysis of potential moderators that might influence the relationship between these learning pathways and AS. For example, as some previous studies have shown that females tend to report higher levels of AS in adult (Stewart et al., 1997) and child samples (Deacon et al., 2002; Muris et al., 2001; Stassart et al., 2014; van Widenfelt et al., 2002; Walsh et al., 2004), including sex as a moderator would allow us to investigate whether the relationship between fear learning pathways and AS differs in male as compared to female children and adolescents. Similarly, including age as a moderator is important, as the salience of specific learning pathways may vary across developmental stages (e.g., parental behaviors such as modeling might be particularly influential during childhood, while other pathways may be more prominent in adolescence) (for a review, see Allen et al., 2023; Leen-Feldner et al., 2006). Furthermore, considering ethnicity as a moderator is essential to account for potential cultural variations in the experience and expression of AS, as well as in the type of learning pathways that may be most salient. For example, cultural norms around emotion expression or parenting practices may influence the relationship of learning pathways and the AS (Cervantes, 2002; Essau et al., 2008, 2011; Graham & Weems, 2014; Ho et al., 2018; Varela et al., 2009).

The primary aim of the present review was to synthesize and combine the available evidence from studies investigating the relationship between parent-to-child fear learning pathways and AS. By doing so, this review aims to gain an in-depth understanding of how and which fear learning pathways may be associated with these fears, which, in turn, may help inform prevention programs and improve existing treatment strategies, for example, by targeting and preventing AS in at-risk children and adolescents (Bernstein & Zvolensky, 2007; Sherman et al., 2019).

## Method

The present systematic review was designed and reported in accordance with the guidelines published in the Preferred Reporting Items for Systematic Reviews and Meta-Analyses (PRISMA) statement (Page et al., 2021).

### Search Strategy

To identify all the relevant articles, a systematic literature search was performed on December 4, 2023 using four electronic databases: PubMed, Web of Science, PsycINFO, and Embase. The search was limited to journal articles investigating past or present parent-to-child learning experiences in the context of anxiety-related symptoms. The literature search was restricted to publications in English language, with an unrestricted date of publication but limited up to the search date on December 4, 2023. The selection of the search terms in this systematic review was based on the preliminary literature search and in consultation with references embedded in previously published reviews on the similar topic (e.g., Askew & Field, 2008; Fisak & Grills-Taquechel, 2007; Lebowitz et al., 2016; Nimphy et al., 2023). The first two authors (EA and JG) conducted the search by using the search terms for each database separately (see “Appendix A” for an overview of the search terms used).

### Eligibility Criteria

The eligibility criteria for this systematic review were developed in accordance with the PICOS framework [(a) Population, (b) Intervention, (c) Comparison, (d) Outcome, (e) Study type] (Methley et al., 2014). To be included in the review, published articles needed to meet the following primary criteria: (a) having human child, adolescent, or adult participants (e.g., aged between 6 and 60 years), with no demographic restrictions, (b) addressing at least one fear learning pathway in the context of anxiety-related symptoms, (c) having no comparison criteria defined, (d) an outcome measure evaluating AS (i.e., Anxiety Sensitivity Index), and (e) having quantitative study design. The eligibility of each article was decided based on the title and abstract, including the full-text screening, where necessary (e.g., if the relevant information was missing from title and abstract).

### Study Selection, Data Extraction, and Coding

We managed all studies using the Rayyan web management tool (Ouzzani et al., 2016). Studies were initially imported from PubMed (627 studies), Web of Science (761 studies), Embase (740 studies), and PsychInfo (688 studies) databases. Titles and abstracts of all studies were screened independently by the first two authors, including the selection and full-text extraction of all included studies based on the criteria described above.

Based on the full-text screening, the first two authors (EA, JG) extracted the following information from the included studies: title, first author, year, sociodemographic information (e.g., age, sex, ethnicity), clinical information (e.g., clinical diagnoses), methodological information (e.g., study location, study design, sample size, measures used for predictor and outcome variables), and statistical information of the variables of interest (e.g., means, standard deviations, correlation coefficients, *p*-values). To be included in the meta-analysis, the statistical information provided by each study had to be sufficient to allow for calculation of effect sizes for the variables of interest. The data were independently extracted and coded by the first and the second author (EA, JG). Overall, there was a 100% agreement between the two authors regarding the extraction of the data from the included studies. Categories were dummy coded for all discrete variables with two levels, including study design type (i.e., retrospective vs. current), sample age group (adults vs. children/adolescents), ethnicity group (homogeneous vs. mixed), and type of learning pathway (vicarious vs. verbal and instrumental).

### Statistical Analyses

Statistical analyses were performed using the ‘metafor’ package in R statistical software (R Core Team, 2022; Viechtbauer, 2010). As most included studies yielded multiple effect sizes, we conducted a three-level meta-analysis, accounting for dependency of clustered effect sizes (Assink & Wibbelink, 2016; Harrer et al., 2021; Viechtbauer, 2010). Correlation coefficients between the fear learning pathways and AS were converted to z-scores using Fisher’s *r*-to-*z* transformation for all analyses and then converted back into correlations using Fisher’s *z*-to-*r* transformation (Borenstein, 2009; DeCoster, 2009). The overall effect was estimated based on the random effects model (Hedges & Vevea, 1998), and restricted maximum-likelihood estimator (REML, Viechtbauer, 2005) was used to calculate *τ*^2^ at level 2 and level 3. Additionally, we used Cochran’s *Q*-test and calculated the *I*^2^-statistic to check for heterogeneity of effects (Cochran, 1954; Hedges & Olkin, 1985; Higgins & Thompson, 2002). Moderators of the overall effect were analyzed using meta-regressions directed by mixed-effects modeling (Hedges & Vevea, 1998).

### Quality Assessment

The National Heart, Lung, and Blood Institute’s Assessment Tool for Observational Cohort and Cross-Sectional Studies (National Heart, Lung, and Blood Institute, 2021) was used to assess the quality of all studies included in this review. The quality assessment tool consists of 14 items that check the internal validity of each study (see “Appendix B” for item descriptions). The first two authors (EA and JG) independently evaluated each of the included studies based on the 14 items, scoring “yes” if the criteria were fully met, “no” if the criteria were not met, or “other” if the criteria could not be determined, were not applied, or were not reported. Based on these scores, we calculated the overall quality rating for each study by summing up the number of items scoring “yes” for each study. Total score ranged from 0 to 14, with studies being assigned either poor, fair, or good rating. Studies obtaining less than 50% of the total score were assigned a poor rating, studies obtaining between 50 and 75% of the total score were assigned a fair quality rating, and studies obtaining above 75% of the total score were assigned a good quality rating. If the overall quality rating of a study was either fair or good, this study was considered to have satisfactory internal validity and less risk of bias.

## Results

**Fig. 1:**
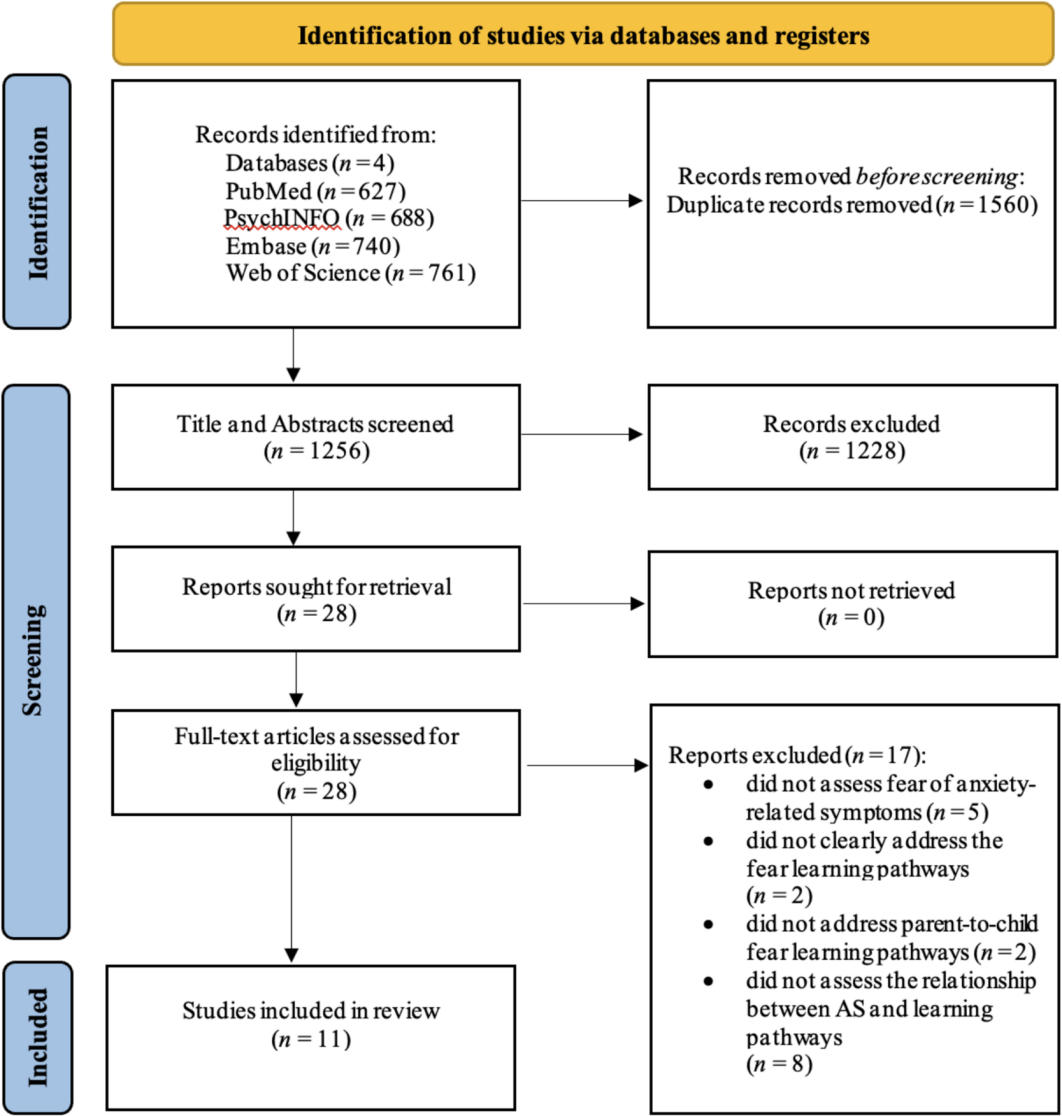
PRISMA flowchart of the systematic search

The initial database search retrieved a total of 2816 articles, which the first author (EA) manually screened, identified, and deleted 1560 duplicate articles. After deleting the duplicates, in total 1256 articles were deemed eligible for title and abstract screening. The first two authors (EA, JG) independently screened titles and abstracts of 1256 unique article entries to check the fit with the eligibility criteria, with discrepancies resolved through discussion and consensus. Interrater reliability on the inclusion of studies based on their abstract and title was high, reaching Cohen’s kappa index of 0.84 (McHugh, 2012). Based on the abstract and title screening, 1228 studies were excluded, leaving 28 studies eligible for full-text screening. After conducting the full-text screening, 17 studies were excluded (see Fig. 1 for exclusion reasons). Overall, 11 articles were included in the systematic review and 10 studies in the meta-analysis. Figure 1 illustrates the flowchart of the selection process resulting in the final number of included articles.

### Quality Assessment

The studies included in this review were rated as having an overall good methodological quality, with most receiving a fair (*n* = 7; see “Appendix B,” studies 1, 4, 6–10) or good (*n* = 4; see “Appendix B,” 2, 3, 5, 11) rating. Of the 7 studies rated as fair, 5 obtained 72% of the total score, indicating good internal validity despite the fair rating (see “Appendix B,” studies 1, 4, 7, 9, 10). The included studies demonstrated methodological strength in their clearly stated research objectives, specified and defined population, and clearly defined, reliable exposure, and outcome measures. The most common methodological limitation in all included studies was due to the cross-sectional design (e.g., measurement of variables at one time point, and lack of follow-up assessment after the initial variables were measured). Another limitation was that none of the included studies provided a description of the power or a justification of the sample size. Only one study reported that 50% of eligible individuals participated, while most studies did not report participation rates (see “Appendix B,” study 6).

### Overview of the Included Studies

#### Study Design

Out of 11 included studies, all the studies had a cross-sectional design (*n* = 11; see “Appendix D,” studies 1–11), with six studies utilizing a retrospective questionnaire to assess past learning experiences in adults (*n* = 6; see “Appendix D,” studies 1–5, 11).

#### Location of Studies

The present systematic review included studies conducted primarily in the United States (*n* = 4; see “Appendix D,” studies 5, 8, 9, 11) and Canada (*n* = 4; see “Appendix D,” studies 1–4), with additional studies conducted in the Netherlands (*n* = 2; see “Appendix D,” studies 6, 7) and Belgium (*n* = 1; see “Appendix D,” study 10).

#### Sample Characteristics

The review included studies with sample sizes ranging from 52 to 543 participants, totaling 2297 participants across all included studies. Of these, 1632 were adults (947 females), 665 were children and adolescents (345 females). Six studies included adult participants, whereas five studies primarily consisted of child or adolescent samples. Of the 11 studies included, only one had a clinical sample (*n* = 1; see “Appendix D,” study 9), while the remaining studies were primarily consisting of non-clinical samples (*n* = 10).

#### Measures

The assessment of the fear learning pathways was based on the Learning History Questionnaire (LHQ; Ehlers, 1993) or its modified versions (*LHQ-expanded version*, Watt et al., 1998; *LHQ-Revised*, Watt & Stewart, 2000; *LHQ-III*, Stewart et al., 2001; Leen-Feldner et al., 2008; Knapp et al., 2013; *LHQ-IV*, Watt et al., 2008; *LHQ-modified*, McGinn et al., 2015; *Parental socialization of anxious behaviors interview schedule (P-SABIS)*, Holly & Pina, 2015) across the included studies assessing childhood learning experiences in adults (*n* = 6; see “Appendix D,” studies 1–5, 11). Two other studies have employed the Learning Experiences Interview (LEI), an adapted version of the LHQ, to evaluate childhood (present) learning experiences in children and adolescents (see “Appendix D,” studies 6, 7), whereas one study has used Learning Experience Index-second version (LEI-II), which is an expanded version of LEI (Stassart et al., 2017; see “Appendix D,” study 10).

For the assessment of fear of anxiety-related symptoms, six included studies used the Anxiety Sensitivity Index (ASI; Reiss et al., 1986) or its modified versions (ASI-Revised, Taylor & Cox, 1998; ASI-3, Taylor et al., 2007) in adult populations (see “Appendix D,” studies 1–5, 11). The ASI and its modified versions have strong psychometric properties, including internal consistency, test–retest reliability, criterion validity, convergent, and discriminant validity (Allan et al., 2014; Dehon et al., 2005; Reiss et al., 1986; Rifkin et al., 2015; Rodriguez et al., 2004; Taylor et al., 2007; Vujanovic et al., 2007). Although the Anxiety Sensitivity Index is often used as a unidimensional measure (Asmundson et al., 2011), previous studies have found multidimensional latent structure, identifying three, lower-order factors (i.e., physical, social, and cognitive domains), which load onto a single, higher-order, general factor (Asmundson et al., 2011; Calamari et al., 2008; Dehon et al., 2005; Farris et al., 2015; Taylor et al., 2007; Vujanovic et al., 2007; Walsh et al., 2004). In the present review, 4 out of 6 included studies reported an overall ASI score (see “Appendix D,” studies 1, 2, 5, 11) and only two studies reported on other ASI facets, such as its physical, cognitive, and social domains (see “Appendix D,” studies 3, 4).

The remaining five included studies used Child Anxiety Sensitivity Index (CASI; Muris, 2002; Silverman et al., 1991) to assess fear of anxiety-related symptoms in children and adolescents. The CASI has also demonstrated strong psychometric properties, including internal consistency, criterion, concurrent, and discriminant validity (Kearney et al., 1997; McLaughlin et al., 2007; Muris et al., 2001; Rabian et al., 1999). Some previous studies have found multidimensional factor structure for CASI (Adornetto et al., 2008; Bernstein & Zvolensky, 2007; Essau et al., 2010; Lambert et al., 2004; Muris et al., 2001; Silverman et al., 2003; Weems et al., 2002, 2010), however, in the present review, studies have only relied on reporting of the overall CASI score (see “Appendix D,” studies 6–10).

### Main Results

#### Systematic Review

**Fig. 2:**
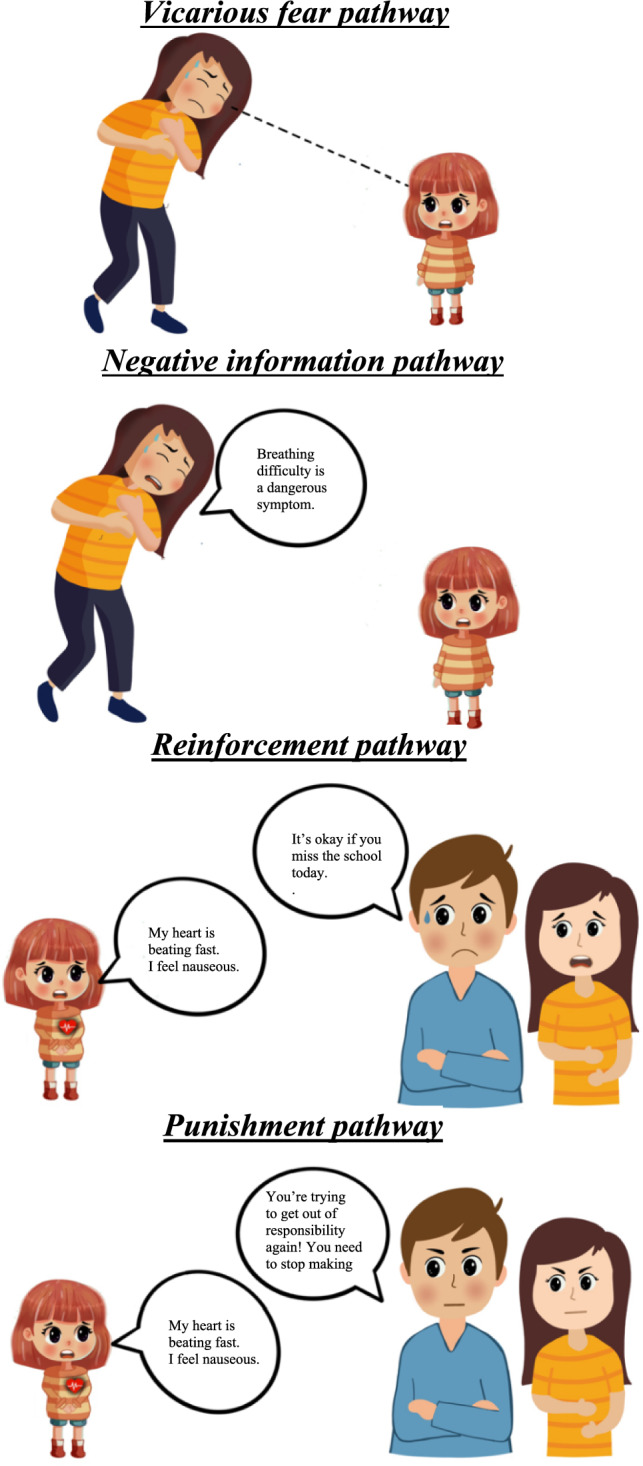
Illustration of fear learning pathways. Illustration of vicarious fear pathway, in which the child observes the fear reaction of a parent experiencing anxiety symptom (e.g., difficulty breathing). Illustration of negative information pathway, in which the parent verbally transmits threat information about anxiety symptoms by informing the child about their fear and belief about the dangerousness of anxiety symptoms. Illustration of parental reinforcement of anxious behavior (e.g., parents supporting their child in avoiding responsibilities such as going to school and giving child attention by providing special treatment in response to child’s complaint such as nausea or heart palpitations). Illustration of parental punishment of anxious behavior (e.g., parents not taking their child’s complaints seriously and showing disapproval)

Table in the “Appendix D” displays a summary of the main findings from the included studies. Given that all studies used a cross-sectional design, the main findings are categorized according to the temporal aspects of the collected data (retrospective versus present). Thus, studies that examine participants’ past learning experiences, such as those that rely on adults’ reports of their childhood learning history, are classified as *retrospective learning experience studies*. In contrast, studies that focus on the ongoing or most recent learning experiences of children and adolescents are categorized as *present learning experience studies*. In addition, as the majority of the included studies combined negative information transmission and parental reinforcement/punishment in their analyses, these pathways are also combined in the main results to maintain consistency and facilitate comparison across studies (see Fig. 2).

##### Vicarious Fear Pathway

*Retrospective learning experience studies.* The relationship between vicarious learning experiences and the AS was examined in 6 studies, in which childhood recollection of vicarious learning from parents was assessed by means of retrospective questionnaires (see “Appendix D,” studies 1–5, 11). These studies collectively found that adults with heightened AS retrospectively reported more experiences involving observation of parental fear reactions in response to anxiety-related symptoms (McGinn et al., 2015; Stewart et al., 2001; Watt & Stewart, 2000; Watt et al., 1998, 2008). Only one study did not find a significant correlation between vicarious fear learning pathway and AS (Leen-Feldner et al., 2008; see “Appendix D,” study 11). Two studies examined the relationship between vicarious fear pathway and the physical, social, and cognitive components of AS (see “Appendix D,” studies 3 and 4). Both studies found a significant correlation with the physical component of AS. However, the results were mixed for the cognitive and social components. One study reported a significant association with the cognitive component but not the social (Watt et al., 2008), while the other found a significant association with the social component but not the cognitive (Stewart et al., 2001).

*Present learning experience studies.* Overall, 3 studies investigated the relationship between children’s and adolescents’ current vicarious fear learning experiences and AS (see “Appendix D,” studies 6, 7, 10). Out of 3, one study found that this pathway was positively associated with AS levels (Stassart et al., 2017), whereas another did not find a significant correlation between vicarious fear learning and adolescents’ AS (Muris et al., 2001). An additional study assessed vicarious fear learning pathway and AS but did not report correlation between these two variables (Muris & Meesters, 2004).

##### Negative information, Reinforcement, and Punishment

*Retrospective learning experience studies.* The fear learning pathway based on negative information, reinforcement, and punishment has been investigated in six studies (see “Appendix D,” studies 1–5, 11), by asking adults to recall when their parents shared **negative information** (e.g., “Did your parents warn you of the possible dangers of your symptoms?”), **negative reinforcement** (e.g., “When you had these symptoms prior to age 18, did your parent encourage you to stay home from school? Were you excused from doing school homework?”), **positive reinforcement** (e.g., “Did you receive special care? Were you allowed to do things which were otherwise not allowed, such as watching television or staying up late?”), and **parental punishment** (“Were you made to feel responsible for having caused your symptoms? Did you feel left alone?”). The findings from these studies revealed a significant association between these childhood fear learning experiences and AS, suggesting that parents’ transfer of negative information about anxiety symptoms, reinforced behaviors, or administered punishment are associated with the elevated levels of AS in adulthood (Leen-Feldner et al., 2008; McGinn et al., 2015; Stewart et al., 2001; Watt & Stewart, 2000; Watt et al., 1998, 2008). Two studies examining the association between negative information, reinforcement, and punishment on various aspects of AS (see “Appendix D,” studies 3 and 4) found significant correlations for the physical and social dimensions of AS. However, results were mixed for the cognitive dimension, with one study (Watt et al., 2008) showing a significant association, while the other (Stewart et al., 2001) did not.

*Present learning experience studies.* Five studies assessed present learning experiences by asking children and adolescents to report on their parents’ verbal expressions of the idea that anxiety-related symptoms might be dangerous (e.g., ‘Do your parents warn you of the possible dangers of your anxiety symptoms?’), parental reinforcement (e.g., ‘When you have these symptoms, do your parents allow you to stay home from school?’), and parental punishment (e.g., ‘When I experience these bodily symptoms, my mother will not take me seriously’) (see “Appendix D,” studies 6–10). Of these five studies, four reported a significant correlation between these learning experiences and anxiety sensitivity (AS) (Knapp et al., 2013; Muris et al., 2001; Stassart et al., 2017). Out of five, one study found that clinic-referred children and adolescents with these experiences also tend to have greater levels of AS (Holly & Pina, 2015). This study investigated the relationship between these learning experiences and AS in a sample of both Caucasian and Hispanic/Latino children and adolescents, finding a significant correlation between these fear learning pathways and AS in both groups. Another study investigated the relationship between parental sharing of negative information, reinforcement, punishment, and AS but did not report correlations between these variables (Muris & Meesters, 2004). 

### Meta-analysis

**Fig. 3:**
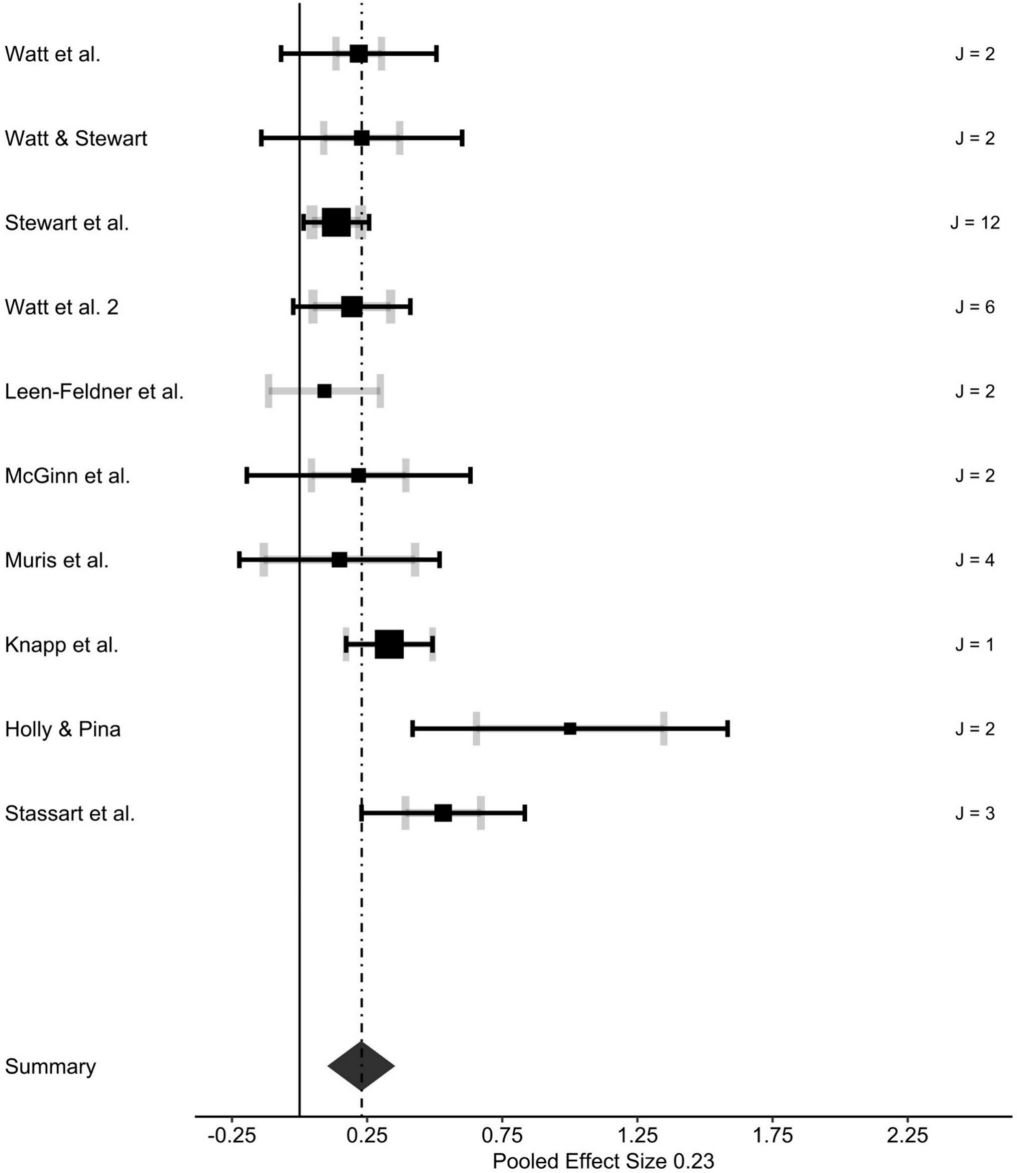
Forest plot of effect sizes

The estimated overall effect across all included studies (*k* = 10) and effect sizes (*u* = 36) was *r* = 0.24, *p* < 0.01, 95% CI [0.13–0.35], indicating a small, but significant relationship between learning experiences and AS (see Fig. 3 for forest plot). Heterogeneity across the studies was not significant (*Q*_35_ = 15.190, *p* = 0.999). Heterogeneity at level 3 (between studies) was at least small as indicated by *I*^2^-_level 3_ = 36.94% (Higgins, 2003). Despite minor variation of the true effect between studies, we employed moderator analyses to assess the effect of essential study-level variables on differences in effects (see “Appendix C”).

Our moderator analyses did not yield a significant moderating effect of learning pathways (*F* = 1.054, *B* = − 0.088*, *SE = 0.086*, p* = 0.312, *df* = 33). The type of study design, however, significantly moderated the relationship between learning experiences and AS (*F* = 6.31, *B* = 0.285*, *SE = 0.113*, p* = 0.0169, *df* = 34). For studies assessing past learning experiences, using retrospective questionnaires, the overall association was smaller, but still statistically significant (*r* = 0.16, *t* = 3.481, *p* = 0.001). In contrast, for studies assessing present learning experiences, we found a higher overall effect (*r* = 0.438, *t* = 2.512, *p* = 0.0169), suggesting that the relationship between learning experiences and AS varies depending on whether the learning experiences are assessed retrospectively or not (see “Appendix C”).

Similarly, moderator analyses revealed a significant effect of sample age group (*F* = 6.31, *B* = 0.285*, *SE = 0.113*, p* = 0.017, *df* = 34), indicating a greater effect for children and adolescents (*r* = 0.438, *t* = 2.512, *p* = 0.017), and a lower, but significant effect for adults (*r* = 0.160, *t* = 3.48, *p* = 0.001). While learning experiences do influence AS in adults, the stronger effect in children and adolescents suggests that learning experiences are more strongly associated with AS in younger populations (see “Appendix C”).

The proportion of females within a sample (*F* = 1.617, *B* = − 0.007*, *SE = 0.005*, p* = 0.212, *df* = 34) and the ethnic composition of the sample (*F* = 0.665, *B* = − 0.170*, *SE = 0.209*, p* = 0.423, *df* = 24) did not significantly moderate the relationship between the learning experiences and AS (see “Appendix C”).

## Discussion

The influence of fear learning pathways on the development of childhood fears has been well established in prior systematic reviews and meta-analyses (Askew & Field, 2008; Fisak & Grills-Taquechel, 2007; King et al., 1998; Lebowitz et al., 2016; Nimphy et al., 2023, 2024). However, these reviews have predominantly focused on investigating learning pathways related to fear of external threats, such as novel animals, snakes, or spiders. In contrast, the literature on learning pathways in relation to internal threats, such as fear of anxiety-related symptoms or AS, has not yet been systematically reviewed. To address this gap, the present systematic review and meta-analysis synthesized findings from individual studies examining the relationship between parent-to-child fear learning pathways and anxiety sensitivity (AS). The results revealed significant associations between childhood learning experiences—such as vicarious fear learning, negative information, reinforcement, and punishment, occurring within the family environment—and AS, suggesting that these pathways are also important to consider in the context of AS. The meta-analysis demonstrated a small but significant relationship between all learning pathways and AS (*r* = 0.24, *p* < 0.01, *k* = 10, *u* = 36), further confirming that these pathways extend beyond external to internal threats.

While individual studies have demonstrated associations between fear learning pathways and AS, they have often yielded inconsistent findings due to variations in methodology, sample characteristics, and study design. By systematically reviewing and aggregating findings across individual studies, our meta-analysis not only consolidates these findings into a single, more robust estimate but also reveals that the impact of these learning pathways is significantly moderated by age and study design, providing novel insights into when and how these pathways may be most influential. Importantly, our results suggest that childhood and adolescence may represent sensitive periods during which fear learning pathways exert stronger effects, supporting the need for early interventions. This quantitative synthesis also helps clarify previous inconsistencies by demonstrating that assessments of present learning experiences produce stronger effects than retrospective reports of learning experiences, emphasizing the need for methodological improvements in future research. Each of the key findings and the implications for theory and intervention are discussed below.

One key finding of our meta-analysis was that the relationship between fear learning pathways and AS varied depending on how learning experiences were assessed. Studies that examined present learning experiences found a stronger association with AS compared to those relying on retrospective reports of childhood learning experiences. Although cross-sectional studies relying on retrospective questionnaires are an important source of evidence that have confirmed the significant association between fear learning pathways and AS, they also come with limitations. One potential limitation of retrospective studies is their reliance on participants’ recall of past learning experiences, which may introduce bias or inaccuracies due to memory distortions or the subjective interpretation of past events. This finding suggests that future research should prioritize assessments of fear learning pathways using longitudinal research designs, as they may offer a more accurate representation of how fear learning pathways might be related to AS. Additionally, these results have implications for clinical interventions, highlighting the importance of focusing on present learning experiences in therapeutic settings to prevent and mitigate AS in children and adolescents.

Our meta-analytic results also revealed that age significantly moderates the relationship between fear learning pathways and AS, with stronger associations in children and adolescents compared to adults. This finding is not surprising as it aligns with developmental research suggesting that children and adolescents are particularly reliant on their parents to interpret and respond to their environment (Allen et al., 2023). Given their still-developing cognitive and emotional regulation skills, children and adolescents may be more reliant on parental behavioral cues to assess whether bodily symptoms are benign or threatening. Alternatively, it is possible that learning experiences reported by children and adolescents are more recent and emotionally salient, making parental modeling of fear, verbal information about threats, and parental reinforcement or punishment more impactful. In contrast, adults who have retrospectively reported on their childhood learning experiences may have reframed those experiences, potentially weakening the relationship between childhood fear learning pathways and their current AS levels. Thus, the age at which learning experiences occur is an important factor to consider in future research investigating parent-to-child fear learning pathways and AS.

Interestingly, our meta-analysis found that the type of fear learning pathway did not significantly moderate the relationship between learning pathways and AS. This may suggest that these pathways do not operate independently but rather co-occur and amplify each other’s impact. For example, a child who observes a parent reacting fearfully to anxiety symptoms may also receive verbal warnings about the dangers of those symptoms. This interpretation aligns with existing fear acquisition models, which posit that fear learning pathways do not operate in isolation in the context of external threats (Field, 2006; Field & Purkis, 2011; Field & Storksen-Coulson, 2007; Mineka & Zinbarg, 2006; Rachman, 1977). However, measurement inconsistencies across reviewed studies (e.g., some studies combining pathways rather than assessing them separately) may have made it difficult to isolate the unique contribution of each pathway to AS. Another likely explanation is the limited number of studies examining each learning pathway separately, resulting in insufficient power to detect significant effects. Thus, future research should aim to assess each pathway individually or utilize standardized measures that better distinguish between these pathways in relation to AS.

In contrast to study design and age, gender and ethnicity did not significantly moderate the relationship between fear learning pathways and AS. While some prior studies have found gender differences in AS levels in adult and youth populations (Deacon et al., 2002; Muris et al., 2001; Stewart et al., 1997; van Widenfelt et al., 2002; Walsh et al., 2004), our findings suggest that the fear learning pathways related to AS may operate similarly across males and females. Likewise, our results indicate that the association between fear learning pathways and AS is relatively comparable across ethnic groups. However, given that the studies in our meta-analysis were predominantly conducted in Western populations, future research should examine fear learning pathways in more diverse and representative samples. Expanding research to non-Western contexts would help clarify whether sociocultural factors may influence the relationship between fear learning pathways and AS.

Although our review largely supports the relationship between fear learning pathways and AS, some studies have reported discrepant findings across pathways. For example, one study found a non-significant correlation between vicarious fear learning and adolescents’ AS but observed significant associations for negative information, reinforcement, and punishment (Muris et al., 2001). Developmental research on AS has suggested that younger children, compared to adolescents, may lack the cognitive capacity to interpret anxiety-related symptoms in a catastrophic manner (Chorpita & Lilienfeld, 1999; Nelles & Barlow, 1988). However, other studies indicate that while younger children can recognize anxiety symptoms, they may struggle to make internal attributions or provide logical reasoning for these symptoms (Muris et al., 2008, 2010b; Weems et al., 1998, 2021). Given their greater reliance on parental behavioral cues, younger children may be particularly susceptible to vicarious fear learning pathway, whereas adolescents, with their more advanced cognitive capacities, may be more influenced by pathways such as negative information or reinforcement (Allen et al., 2023). However, given considerable age variability across the reviewed studies, with some studies focusing only on children, others only on adolescents, and some combining both age groups, future research should investigate whether different fear learning pathways vary in their influence at different developmental stages.

One striking pattern across our review was that most included studies relied on cross-sectional methodologies, in non-clinical populations, to investigate the relationship between fear learning pathways and AS. While these studies provide valuable insights into the relationship between fear learning pathways and AS, they cannot establish causality. This contrasts with previous research using experimental paradigms to investigate and demonstrate that parent-to-child learning pathways contribute to the onset of fear in response to external threats (e.g., snakes, strangers) (Burstein & Ginsburg, 2010; De Rosnay et al., 2006; Dubi et al., 2008; Gerull & Rapee, 2002; Marin et al., 2020; Muris et al., 2010a). Such findings align with contemporary fear acquisition models, which suggest that fear learning pathways operate through associative learning mechanisms (Aktar & Pérez-Edgar, 2024; Askew & Field, 2008; Field, 2006; Olsson & Phelps, 2007; Mineka & Cook, 1993; Mineka & Zinbarg, 2006). For example, in vicarious fear learning, a recent study using vicarious conditioning paradigm demonstrated that children could acquire fear of a previously neutral conditioned stimulus (CS) after observing a parent’s fearful reaction to it. The parent’s fearful reaction serves as an unconditioned stimulus (US), leading the child to associate the previously neutral stimulus with threat and subsequently exhibit a conditioned fear response (Marin et al., 2020; Dunne & Askew, 2013; for a review, see Skversky-Blocq et al., 2021). While this research provides compelling evidence for the role of vicarious learning in fear acquisition, no studies to date have used conditioning paradigms to examine whether similar processes occur in the context of anxiety-related symptoms. This gap highlights the need for experimental research to investigate children’s and adolescents’ fear acquisition of anxiety-related symptoms through these learning pathways.

Although the included studies provide evidence that parent-to-child fear learning pathways are related to AS, the cross-sectional nature of these studies prevents conclusions about whether these pathways contribute to the onset of AS or shape its trajectory over time. If parental behaviors toward bodily symptoms, through fear learning pathways, increase offspring’s fear of these symptoms, they may also reinforce avoidance, potentially contributing to the maintenance of AS (Lebowitz et al., 2016; Bunaciu et al., 2014; Weems et al., 2002). However, given the lack of longitudinal studies, this remains a critical gap, particularly since both AS and parental behaviors have been implicated in the onset and persistence of anxiety-related symptoms.

While the includes studies in our review have focused on how parental behaviors are related to child AS, emerging evidence suggests that this relationship may be bidirectional, with children’s AS potentially influencing parental behaviors via these fear learning pathways. For instance, two experimental studies have shown that adolescents who describe their bodily symptoms in an anxious manner elicit greater parental reinforcement behaviors in response to those symptoms, such as encouraging rest or avoidance (e.g., telling the child to lie down) (Bilsky et al., 2018a, b). These findings suggest that child AS-related distress influences parental behaviors via reinforcement pathway, which may inadvertently reinforce avoidance, thereby creating a vicious cycle that could maintain AS over time. Future longitudinal research is needed to explore whether reinforcement or other fear learning pathways contribute to AS maintenance and, if so, whether interventions targeting these parental responses could help disrupt this cycle.

### Conceptual Model

**Fig. 4:**
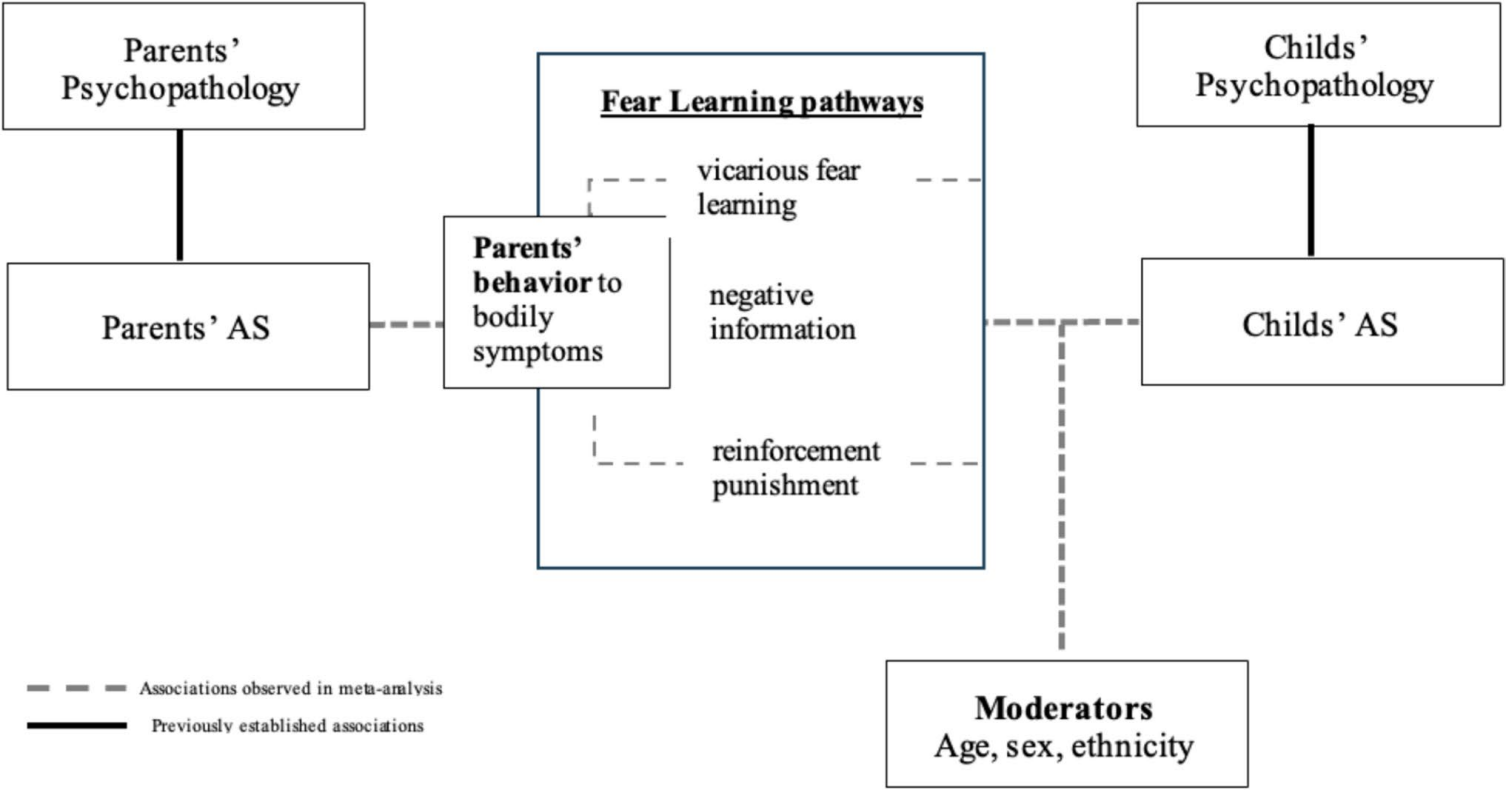
Conceptual model illustrating associations between parent-to-child learning pathways and anxiety sensitivity (AS). Solid lines represent significant associations identified in previous research, such as the link between parents’ AS and their own psychopathology (Olatunji & Wolitzky-Taylor, 2009; Schmidt et al., 2006; Taylor et al., 1996). The significant relationship between AS and subsequent psychopathology in children is also depicted in the upper right corner of the figure (Noël & Francis, 2011; Weems et al., 1997). Dashed lines represent associations observed in the current meta-analysis, which found a significant relationship between parent-to-child fear learning pathways (e.g., vicarious fear, negative information, and reinforcement/punishment) and AS. The strength of these associations varied based on the recency of reported learning experiences and offspring age, with stronger associations observed in younger populations (children and adolescents). However, gender and ethnic composition did not significantly moderate these associations

In sum, our findings highlight that childhood learning experiences, such as vicarious learning, negative information transmission, reinforcement, and punishment, are significantly and positively correlated with fear of anxiety-related symptoms or AS (see Fig. 4). Based on the available evidence and the findings of our meta-analysis, we developed an initial conceptual model to illustrate how fear learning pathways are related to the AS (see Fig. 4). As shown in Fig. 4, this model highlights established significant associations supported by prior research in solid lines, whereas associations supported by the current meta-analysis are shown in dashed lines. More specifically, a significant association between parents’ AS and parents’ psychopathology has been demonstrated, with previous research showing that elevated AS levels are associated with subsequent anxiety and depressive disorders in adults (Naragon-Gainey, 2010; Hovenkamp-Hermelink et al., 2019; Olatunji & Wolitzky-Taylor, 2009; Schmidt et al., 2006; Spinhoven et al., 2017). Similar to findings reported for adults, AS has been found to serve as a pertinent risk marker for subsequent anxiety psychopathology in children and adolescents (Hale & Calamari, 2006; Noël & Francis, 2011; Weems et al., 1997, 2002). Although AS is correlated with anxiety symptoms and later psychopathology in adult and child population (Joiner et al., 2002; Schmidt et al., 1997, 1999; Tsao et al., 2005), the parent-to-child fear learning pathways related to AS have not been fully elucidated. The present meta-analysis provided evidence for the significant association between the learning pathways and AS, suggesting that exposure to such learning experiences, whether through verbal communication, modeling, or reinforcement/punishment, is related to AS. The relationship between learning pathways and AS was found to be stronger when current learning experiences were assessed, indicating that recent interactions may have a more immediate and impactful role in shaping AS. Moreover, this relationship appeared to be more pronounced in younger populations, such as children and adolescents, compared to adults. These findings suggest that developmental stages may influence the extent to which individuals are susceptible to learning pathways, reinforcing the importance of considering age when evaluating the learning pathways in the context of AS. However, the moderating effects of gender and ethnic composition were not significant, indicating that these demographic variables may not substantially alter the relationship between learning pathways and AS. Overall, these findings suggest that further investigation of pathways through which fear of anxiety-related symptoms or AS is learned is necessary, including the need for targeted preventive interventions that address AS via these specific fear learning pathways. Future research should prioritize longitudinal and experimental methodologies to further investigate these relationships and develop interventions that can ameliorate the risk of developing anxiety-related psychopathology.

### Clinical Implications

A deeper understanding of the learning experiences involved in relation to AS may inform and significantly improve the existing treatment interventions and prevention strategies, while also leading to the development of targeted prevention programs designed particularly to address fear learning of anxiety symptoms. In our meta-analysis, we found a small but significant association between learning pathways and AS, demonstrating that vicarious learning experiences, verbal learning experiences, and parental reinforcing and punishing behaviors during childhood and adolescence are all significantly associated with elevated levels of AS. Given these findings, parent-based treatment interventions should aim to target all these learning pathways. It could be possible that by addressing each pathway in an integrated manner, the efficacy of interventions may be enhanced, thereby potentially preventing later onset of anxiety sensitivity.

At present, however, preventive interventions and specific programs targeting these learning pathways and additionally addressing AS are lacking. In fact, there are only a few intervention studies aimed at preventing the transmission of anxiety in the offspring of anxious parents (Cartwright-Hatton et al., 2018; Ginsburg et al., 2015, 2020). However, these intervention studies have demonstrated effectiveness in reducing anxiety by modifying fear and anxiety-enhancing parental behaviors (for a review, see Chapman et al., 2022). For example, parent-based interventions targeting parent-to-child interactions, such as providing psychoeducation to reduce parental modeling of anxious behaviors, have shown positive outcomes in reducing the overall anxiety symptoms and decreasing rates of onset of anxiety disorders in children of families receiving such intervention (Cartwright-Hatton et al., 2018; Ginsburg et al., 2015, 2020).

Additionally, some interventions addressing somatic symptoms also exist, but these have primarily focused on the augmentation of child therapy, including cognitive-behavioral therapy, with a family-based component (for a review, see Coakley & Wihak, 2017; Levy et al., 2010; Zagustin, 2013). For example, one intervention study that has addressed social learning strategies to modify family responses to illness and wellness behaviors, showed reduction in pain and gastrointestinal symptoms in children and adolescents after parents reduced protective responses to children’s symptoms and decreased maladaptive beliefs related to children’s pain (Levy et al., 2013; Levy et al., 2010).

Adapting techniques used in these prevention studies, particularly those aimed at reducing the adverse effects of learning pathways, could also be efficient in preventing or reducing AS levels. Future interventions should consider incorporating learning pathways as an added component, teaching parents how their modeling, verbal messages, reinforcement, or punishment may increase children’s fear of anxiety symptoms. In fact, current treatment and future preventive intervention programs should train parents how to identify and modify these behaviors and teach parents to implement positive modeling, communicate positive information regarding bodily symptoms, and refrain from reinforcing (e.g., special treatment, treats) or punishing behaviors.

Overall, findings from intervention studies suggest that parent-based interventions could be delivered as a stand-alone, alternative prevention strategy (with parents as primary agents of behavioral change), especially in cases where child treatment is not feasible (e.g., younger children).

### Limitations and Future Directions

Although the present systematic review and meta-analysis provide a comprehensive overview of the literature elucidating the role of learning pathways and fear of anxiety symptoms, the following limitations should be considered.

First, given that all the included studies employed a cross-sectional design, the limitation of the present review is that the cross-sectional nature of the reviewed studies and the lack of longitudinal or experimental studies prevent us from drawing any causal conclusions. An additional methodological limitation is the use of retrospective questionnaires in several included studies, due to the memory bias that is inherent in retrospective recall of learning experiences (e.g., adults reporting on their childhood learning experiences). To address such methodological limitations, future studies should employ either longitudinal designs or experimental paradigms to investigate learning of fear of anxiety symptoms.

Second, another limitation of the review is the exclusion of other anxiety-related constructs and measures that might have been related to the measures of interest (i.e., fear of anxiety-related symptoms and learning pathways) but were beyond the scope of this review. Given that our results primarily rely on the Anxiety Sensitivity Index as a measure of fear of anxiety-related symptoms, other constructs or measures that might be related might have been excluded. Future studies should also aim to include a broader range of anxiety-related constructs and measures, beyond just anxiety sensitivity, to explore how other dimensions of anxiety may interact with these learning pathways. By examining constructs such as trait anxiety, intolerance of uncertainty, and worry, a broader insight might be uncovered regarding mechanisms that could contribute to the development of fear of anxiety symptoms. Incorporating these constructs could provide a more nuanced understanding of how various fear-related constructs (e.g., fear of anxiety symptoms, intolerance of distress) intersect with learning experiences, potentially offering more comprehensive insights for interventions targeting anxiety psychopathology.

Third, the results presented in our review and meta-analysis relied primarily on the studies conducted in North America and Western Europe. Additionally, the samples consisted predominantly of Caucasian participants, with a few studies including Hispanic/Latino, Black/African American, or Asian participants. Given that previous research shows that anxiety sensitivity is linked to anxiety symptoms and disorders in heterogeneous racial and ethnic adult (Ghisi et al., 2016; Lim & Kim, 2012; Sandín et al., 2007; Talkovsky & Norton, 2014; Zvolensky et al., 2003) and youth population (Chorot et al., 2023; Essau et al., 2008, 2011; Fernández-Valdés et al., 2017; Fullana et al., 2003; Lambert et al., 2004), the homogeneity of samples in studies included in our review limits representatives and generalizability of our findings. Thus, future studies would benefit from investigating the role of learning pathways and anxiety sensitivity in a more demographically inclusive and culturally diverse population. However, considering that we limited our search to peer-reviewed studies published only in English, we may have missed other relevant studies published in other languages, as well as those using different search terms or databases, which could have provided increased generalizability of the findings presented in this review.

A notable limitation of the present review is the reliance on studies that predominantly reported the overall anxiety sensitivity scale, rather than its multidimensional subscales (e.g., physical social, cognitive). Based on our systematic search, only two reviewed studies specifically assessed different facets of AS, producing mixed results. These discrepancies suggest that the relationship between learning pathways and the specific facets of AS may be more complex than initially thought. Additionally, the reliance on the overall anxiety sensitivity and the absence of data on specific facets of AS limit our ability to draw conclusions on the relationship between these learning experiences and different facets of AS. Future research should aim to investigate these facets more thoroughly, employing larger sample sizes and longitudinal designs to clarify whether different learning pathways uniquely contribute to each component of AS. By doing so, future research may provide more nuanced insights with important implications for developing more targeted interventions that address the distinct elements of anxiety sensitivity.

## Appendix A

Search terms applied to four electronic databases: PubMed, Embase, PsychINFO, Web Science

**Table:**

Search category	Search terms
1 *fear learning* *pathways*	Vicarious learning *OR* vicarious fear learning *OR* observational learning *OR* observational fear learning *OR* social learning *OR* social fear learning *OR* social fear transmission *OR* modeling *OR* modeling *OR* informational learning *OR* instructed fear learning *OR* verbal information *OR* verbal threat information *OR* learning exp* *OR* learning history *OR* reinforcement learning *OR* punishment *OR* instrumental learning
	*AND*
2 *anxiety-related* *symptoms*	Anxiety sensitivity *OR* anxiety symptom* *OR* anxiety-related symptom* *OR* anxiety sensation* *OR* bodily symptom* *OR* bodily sensation* *OR* physical symptom* *OR* physical sensation* *OR* somatic symptom* *OR* somatic sensation* *OR* panic *OR* panic symptom* *OR* panic-related symptom* *OR* panic attack* *OR* arousal reactive sensation* *OR* health anxi* *OR* illness anxi* *OR* illness behavior *OR* illness behaviour *OR* hypochondria* *OR* hypochondriacal worry *OR* somatic-complaint*
	*AND*
3 *population*	Child* *OR* adolesc* *OR* pediatric *OR* paediatric *OR* offspring *OR* youth *OR* young adult *OR* parent* *OR* mother* *OR* maternal *OR* father* *OR* paternal *OR* caregiver* *OR* guardian* *OR* famil* *OR* student*

The Boolean operator OR was used within each search category to combine search terms so that results contain at least one of the terms. The Boolean operator AND combines three search categories so that results contain all the search terms

## Appendix B

Quality rating of the included studies

**Table:**

Authors	Item														Quality rating	%
	1	2	3	4	5	6	7	8	9	10	11	12	13	14		
1. Watt et al. (1998)	Yes	Yes	NR	Yes	No	No	Yes	Yes	Yes	NA	Yes	NA	NA	Yes	Fair	72
2. Watt and Stewart (2000)	Yes	Yes	NR	Yes	No	No	Yes	Yes	Yes	NA	Yes	NA	NA	Yes	Good	81
3. Stewart et al. (2001)	Yes	Yes	NR	Yes	No	No	Yes	Yes	Yes	NA	Yes	NA	NA	Yes	Good	81
4. Watt et al. (2008)	Yes	Yes	NR	Yes	No	No	Yes	Yes	Yes	NA	Yes	NA	NA	Yes	Fair	72
5. McGinn et al. (2015)	Yes	Yes	NR	Yes	No	No	Yes	Yes	Yes	NA	Yes	NA	NA	Yes	Good	81
6. Muris et al. (2001)	Yes	Yes	Yes	Yes	No	No	No	Yes	Yes	NA	Yes	NA	NA	Yes	Fair	54
7. Muris and Meesters (2004)	Yes	Yes	NR	Yes	No	No	No	Yes	Yes	NA	Yes	NA	NA	Yes	Fair	72
8. Knapp et al. (2013)	Yes	Yes	NR	Yes	No	No	No	Yes	Yes	NA	Yes	NA	NA	Yes	Fair	63
9. Holly and Pina (2015)	Yes	Yes	NR	Yes	No	No	No	Yes	Yes	NA	Yes	NA	NA	Yes	Fair	72
10. Stassart et al. (2017)	Yes	Yes	NR	Yes	No	No	No	Yes	Yes	NA	Yes	NA	NA	Yes	Fair	72
11. Leen-Feldner et al. (2008)	Yes	Yes	Yes	Yes	No	No	Yes	Yes	Yes	NA	Yes	NA	NA	Yes	Good	81

Item 1 = Was the research question or objective in this paper clearly stated?; Item 2 = Was the study population clearly specified and defined; Item 3 = Was the participation rate of eligible persons at least 50%?; Item 4 = Were all the subjects selected or recruited from the same or similar populations (including the same time period)? Were inclusion and exclusion criteria for being in the study prespecified and applied uniformly to all participants?; Item 5 = Was a sample size justification, power description, or variance and effect estimates provided?; Item 6 = For the analyses in this paper, were the exposure(s) of interest measured prior to the outcome(s) being measured?; Item 7 = Was the timeframe sufficient so that one could reasonably expect to see an association between exposure and outcome if it existed?; Item 8 = For exposures that can vary in amount or level, did the study examine different levels of the exposure as related to the outcome (e.g., categories of exposure, or exposure measured as continuous variable)?; Item 9 = Were the exposure measures (independent variables) clearly defined, valid, reliable, and implemented consistently across all study participants?; Item 10 = Was the exposure(s) assessed more than once over time?; Item 11 = Were the outcome measures (dependent variables) clearly defined, valid, reliable, and implemented consistently across all study participants?; Item 12 = Were the outcome assessors blinded to the exposure status of participants?; Item 13 = Was loss to follow-up after baseline 20% or less?; Item 14 = Were key potential confounding variables measured and adjusted statistically for their impact on the relationship between exposure(s) and outcome(s)

*NR* not reported, *NA* not applicable

## Appendix C

Meta-regression results of categorical moderators

**Table:**

Effect	*df*	*F*	*B*	*r*	SE	*t*	95% CI
**Overall Effect Size**	35			**0.236****	0.056		[0.127, 0.355]
**Fear Learning Pathways**	33	1.054					
Negative information, reinforcement, punishment				0.283	0.071	4.073	[0.145, 0.436]
Vicarious fear pathway			− 0.088	0.195	0.086	− 1.027	[− 0.263, 0.087]
**Study Design**	34	**6.310****					
Retrospective				0.161	0.046	3.481	[0.067, 0.257]
Present			0.277	0.438	0.113	2.512	[0.054, 0.515]
**Age Group**	34	**6.310****					
Adults				0.160	0.047	3.481	[0.068, 0.257]
Children and Adolescents			0.278	0.438	0.113	2.512	[0.054, 0.515]
**Ethnic Group**	24	0.664					
Homogeneous				0.396	0.169	2.468	[0.068, 0.769]
Mixed			− 0.168	0.228	0.208	− 0.815	[− 0.60, 0.260]

*p* < 0.1, **p* < 0.05, ***p* < 0.01. Significant findings are presented in bold. Fisher *z*-values used in analyses were converted back to *r*-values for presentation

## Appendix D

Detailed study description and summary

**Table:**

#	Study		Study characteristics		Subjects			Results			
	*First author, year*	*Study type/Methods*	*Sample*	*Age group*	*Country/Ethnicity*	*Cases*	*Controls*	*Fear of anxiety-related symptoms*	*Learning pathway*	*Significant result*	
1	Watt et al. (1998)	Cross-sectional/Retrospective questionnaire	Non-clincial	Adults	Canada	*N* = 543 High ASI (*n* = 88) (${M}_{\text{age}}$ = 20.4, SD = 3.0; * f* = 63) Medium ASI (*n* = 112) (${M}_\text{age}$ = 20.8, SD = 3.8; * f* = 70) Low ASI (*n* = 88) (M_age_ = 21.6, SD = 4.6; * f* = 60)	No control	Assessed with ASI Total score	Assessed with LHQ-Expanded	Vicarious, Negative information, reinforcement & punishment of cold and anxiety-related symptoms (LHQ)	High AS reported sig. more reinforcement and vicarious pathway of arousal reactive, arousal non-reactive symptoms vs. low AS group; All part reinforcement/Negative information/punishment of arousal reactive sig. predicted ASI, but not vicarious learning of arousal reactive symptoms
2	Watt and Stewart (2000)	Cross-sectional/Retrospective questionnaire	Non Clincial	Adults and their parents	Canada	students (*N* = 197) (${M}_\text{age}$ = 21.9, SD = 4.6; * f* = 156)	No controls	Assessed with ASI Total score	Assessed with LHQ-Revised	Vicarious fear pathway, Negative information, reinforcement/punishment (LHQ)	Sig. association between AS and learning pathways of arousal reactive and non-arousal reactive symptoms;
3	Stewart et al. (2001)	Cross-sectional/Retrospective questionnaire	Non-clinical	Adults	Canada 71% Caucasian, 17% Asian, 12% Other ethnic group	*N* = 478 (${M}_\text{age}$ = 21, SD = 3; * f* = 317)	No controls	Assessed with ASI-R Total, content scales	Assessed with LHQ-III	Vicarious fear pathway, reinforcement, Negative information, punishment (LHQ)	Learning history of arousal reactive and non-reactive symptoms associated with AS;
4	Watt et al. (2008)	Cross-sectional/Retrospective questionnaire	Non-clinical	Adults	Canada 93% White	*N* = 192 (${M}_\text{age}$ = 19.4, SD = 3.5; * f* = 143)	No controls	Assessed with ASI Total, Subscales	Assessed with LHQ-IV	Vicarious fear pathway, Negative information, Reinforcement/punishment (LHQ)	Sig. predictor of AS was transmission (vicarious, verbal, reinforcement) of anxiety-related symptoms
5	McGinn et al. (2015)	Cross-sectional/Retrospective questionnaire	Non-clinical	Adults (18–77)	USA New York	*N* = 129 (${M}_\text{age}$ = 42.5, SD = 13.2; * f* = 99)	No control	Assessed with ASI Total score	Assessed with LHQ-Modified	Vicarious fear pathway, reinforcement (LHQ)	Parental reinforcement and vicarious fear of anxiety symptoms was not sig. associated with fear if anxiety symptoms
6	Muris et al. (2001)	Cross-sectional	Non-clinical	Adolescents (12–14 years)	Netherlands	*N* = 52 (${M}_\text{age}$ = 12.3, SD = 0.5; * f* = 29)	No controls	Assessed with CASI Total score	Assessed with LEI	Vicarious fear pathway, Negative information, and reinforcement (LEI)	Sig. association between AS and Negative information of pain and anxiety; no sig. association with reinforcement or vicarious fear pathway
7	Muris and Meesters (2004)	Cross-sectional	Non-clinical	Children (8–13 years)	Netherlands, southern part	*N* = 190 (${M}_\text{age}$ = 10.6, SD = 1.0; * f* = 100)	No Control	Assessed with CASI Total score	Assessed with LEI	Reinforcement, Negative information (LEI)	Trait anxiety, AS and Negative information of anxiety sig. predicted somatization symptoms in children
8	Knapp et al. (2013)	Cross-sectional	Non-clinical	Children and Adolescents (10–17 years)	USA 4.4% Hispanic/Latino, 85% White/Caucasian, 5.9% Asian, 7% Black/African American, 7% American Indian, 3.3.% Other	*N* = 153 (${M}_\text{age}$ = 14.66 (SD = 2.18), * f* = 76)	No Control	Assessed with CASI Total score	Assessed with LHQ-III	Negative information, Reinforcement (LHQ-III)	Positive association between AS and negative information reinforcement of arousal reactive symptoms but not arousal non-reactive symptoms
9	Holly and Pina (2015)	Cross-sectional	Clinical	Children and Adolescents (6–16 years)	USA 49% Hispanic/Latino 51% Caucasian	*N* = 70 (${M}_\text{age}$ = 9.86, SD = 2.59; * f* = 35)	No controls	Assessed with CASI Total score	Assessed with P-SABIS	Vicarious fear pathway, Negative information, reinforcement/ punishment (P-SABIS)	For Hispanic/Latino and Caucasian children: reinforcement of anxious emotion expression was sig. associated with AS
10	Stassart et al. (2017)	Cross-sectional	Non-clinical	Children (9–13 years) and their parents (mother & father)	Belgium 100% Caucasian	N = 200 (${M}_\text{age}$ = 10.7, SD = 1.19; * f* = 105) Mothers (*n* = 200) and fathers (*n* = 200) (${M}_\text{age}$ = 42.0, SD = 4.60)	No controls	Assessed with CASI Total score Other factors: child’s femininity/expressive traits and masculine/ emotional traits (CPAQ)	Assessed with LEI-II	Vicarious fear pathway, Negative information, reinforcement (LEI-II)	AS was sig. associated with mothers and fathers modeling and negative information of pain and anxiety-related symptoms Negative information and reinforcement of pain symptoms sig. predicted child’s AS; Mother’s modeling of anxiety symptoms was sig. associated with child’s AS in children with high feminine/expressive traits (+ 1SD); father’s modeling of anxiety symptoms was sig. associated with child’s AS in children with high feminine/expressive (+ 1SD)
11	Leen-Feldner et al. (2008)	Cross-sectional	Non-clinical	Adults	USA 95% Caucasian 2.5%, African American and, 2.5% Asian	*N* = 93 (${M}_\text{age}$ = 23.41, SD = 8.56, *f* = 39)	No controls	Assessed with ASI Total score	Assessed with LHQ-III	Vicarious fear pathway, Negative information, reinforcement, and punishment (LHQ)	

*PD* panic disorder, *AS* anxiety sensitivity, *LHQ* Learning History Questionnaire, *ASI* Anxiety Sensitivity Index, *CASI* Childhood Anxiety Sensitivity Index, *LEI* learning experience interview, *LHQ-III* Learning History Questionnaire-III, *ASI-R* Anxiety Sensitivity Index-Revised, *ASI-3* Anxiety Sensitivity Index-3, *LEI-II* Learning Experience Index-second version (Stassart et al., 2017), *ASI-R* Anxiety Sensitivity Index-Revised, *CPAQ* Children’s personality Attributes Questionnaire, *P-SABIS* parental socialization of anxious behaviors interview schedule

## References

[CR1] Adornetto, C., Hensdiek, M., Meyer, A., In-Albon, T., Federer, M., & Schneider, S. (2008). The factor structure of the childhood anxiety sensitivity index in German children. *Journal of Behavior Therapy and Experimental Psychiatry,**39*(4), 404–416. 10.1016/j.jbtep.2008.01.001 18295743 10.1016/j.jbtep.2008.01.001

[CR3] Aktar, E., Nikolić, M., & Bögels, S. M. (2017). Environmental transmission of generalized anxiety disorder from parents to children: Worries, experiential avoidance, and intolerance of uncertainty. *Dialogues in Clinical Neuroscience,**19*(2), 137–147. 10.31887/dcns.2017.19.2/eaktar 28867938 10.31887/DCNS.2017.19.2/eaktar PMC5573558

[CR2] Aktar, E., & Pérez-Edgar, K. (2024). Family risk factors in the acquisition of anxiety. *Advances in Psychiatry and Behavioral Health,**4*(1), 225–233. 10.1016/j.ypsc.2024.05.016 39629334 10.1016/j.ypsc.2024.05.016 PMC11611299

[CR4] Allan, N. P., Capron, D. W., Lejuez, C. W., Reynolds, E. K., MacPherson, L., & Schmidt, N. B. (2014). Developmental trajectories of anxiety symptoms in early adolescence: The influence of anxiety sensitivity. *Journal of Abnormal Child Psychology,**42*(4), 589–600. 24062146 10.1007/s10802-013-9806-0 PMC4046901

[CR5] Allen, K. B., Tan, P. Z., Sullivan, J. A., Baumgardner, M., Hunter, H., & Glovak, S. N. (2023). An integrative model of youth anxiety: Cognitive-affective processes and parenting in developmental context. *Clinical Child and Family Psychology Review,**26*(4), 1025–1051. 10.1007/s10567-023-00458-z 37819403 10.1007/s10567-023-00458-z

[CR6] Askew, C., & Field, A. P. (2007). Vicarious learning and the development of fears in childhood. *Behaviour Research and Therapy,**45*(11), 2616–2627. 17651688 10.1016/j.brat.2007.06.008

[CR7] Askew, C., & Field, A. P. (2008). The vicarious learning pathway to fear 40 years on. *Clinical Psychology Review,**28*(7), 1249–1265. 10.1016/j.cpr.2008.05.003 18614263 10.1016/j.cpr.2008.05.003

[CR9] Asmundson, G. J. G., & Taylor, S. (2005). *It’s not all in your head: How worrying about your health could be making you sick-and what you can do about it*. Guilford Press.

[CR10] Asmundson, G. J. G., Weeks, J. W., Nicholas Carleton, R., Thibodeau, M. A., & Fetzner, M. G. (2011). Revisiting the latent structure of the anxiety sensitivity construct: More evidence of dimensionality. *Journal of Anxiety Disorders,**25*(1), 138–147. 10.1016/j.janxdis.2010.08.013 20888185 10.1016/j.janxdis.2010.08.013

[CR11] Assink, M., & Wibbelink, C. J. (2016). Fitting three-level meta-analytic models in R: A step-by-step tutorial. *The Quantitative Methods for Psychology,**12*(3), 154–174. 10.20982/tqmp.12.3.p154

[CR12] Bandura, A., & Walters, R. H. (1977). *Social learning theory* (Vol. 1). Prentice Hall.

[CR13] Bernstein, A., & Zvolensky, M. J. (2007). Anxiety sensitivity: Selective review of promising research and future directions. *Expert Review of Neurotherapeutics,**7*(2), 97–101. 17286543 10.1586/14737175.7.2.97

[CR14] Bilsky, S. A., Cloutier, R. M., Bynion, T.-M., Feldner, M. T., & Leen-Feldner, E. W. (2018a). An experimental test of the impact of adolescent anxiety on parental sick role reinforcement behavior. *Behaviour Research and Therapy,**109*, 37–48. 10.1016/j.brat.2018.07.009 30096451 10.1016/j.brat.2018.07.009 PMC6862720

[CR15] Bilsky, S. A., Feldner, M. T., Bynion, T.-M., Rojas, S. M., & Leen-Feldner, E. W. (2018b). Child anxiety and parental anxiety sensitivity are related to parent sick role reinforcement. *Parenting,**18*(2), 110–125. 10.1080/15295192.2018.1444132

[CR16] Borenstein, M. (2009). *Introduction to meta-analysis*. Wiley.

[CR17] Burstein, M., & Ginsburg, G. S. (2010). The effect of parental modeling of anxious behaviors and cognitions in school-aged children: An experimental pilot study. *Behaviour Research and Therapy,**48*(6), 506–515. 10.1016/j.brat.2010.02.006 20299004 10.1016/j.brat.2010.02.006 PMC2871979

[CR18] Bunaciu, L., Leen-Feldner, E. W., Blumenthal, H., Knapp, A. A., Badour, C. L., & Feldner, M. T. (2014). An experimental test of the effects of parental modeling on panic-relevant escape and avoidance among early adolescents. *Behavior Therapy,**45*(4), 517–529. 24912464 10.1016/j.beth.2014.02.011

[CR19] Calamari, J. E., Rector, N. A., Woodard, J. L., Cohen, R. J., & Chik, H. M. (2008). Anxiety sensitivity and obsessive—compulsive disorder. *Assessment,**15*(3), 351–363. 10.1177/1073191107312611 18310595 10.1177/1073191107312611

[CR20] Cartwright-Hatton, S., Ewing, D., Dash, S., Hughes, Z., Thompson, E. J., Hazell, C. M., Field, A. P., & Startup, H. (2018). Preventing family transmission of anxiety: Feasibility RCT of a brief intervention for parents. *British Journal of Clinical Psychology,**57*(3), 351–366. 10.1111/bjc.12177 29575043 10.1111/bjc.12177

[CR21] Cervantes, C. A. (2002). Explanatory emotion talk in Mexican immigrant and Mexican American families. *Hispanic Journal of Behavioral Sciences,**24*(2), 138–163. 10.1177/0739986302024002003

[CR22] Chapman, L., Hutson, R., Dunn, A., Brown, M., Savill, E., & Cartwright-Hatton, S. (2022). The impact of treating parental anxiety on children’s mental health: An empty systematic review. *Journal of Anxiety Disorders,**88*, 102557. 10.1016/j.janxdis.2022.102557 35397376 10.1016/j.janxdis.2022.102557

[CR23] Chorot, P., Valiente, R. M., & Sandín, B. (2023). The Spanish version of the childhood anxiety sensitivity index: Factorial dimensions and invariance across gender in a sample of adolescents. *International Journal of Environmental Research and Public Health,**20*(4), 3045. 10.3390/ijerph20043045 36833740 10.3390/ijerph20043045 PMC9958708

[CR25] Chorpita, B. F., Albano, A. M., & Barlow, D. H. (1996). Child anxiety sensitivity index: Considerations for children with anxiety disorders. *Journal of Clinical Child Psychology,**25*(1), 77–82. 10.1207/s15374424jccp2501_9

[CR24] Chorpita, B. F., & Lilienfeld, S. O. (1999). Clinical assessment of anxiety sensitivity in children and adolescents: Where do we go from here? *Psychological Assessment,**11*(2), 212–224. 10.1037/1040-3590.11.2.212

[CR26] Cochran, W. G. (1954). The combination of estimates from different experiments. *Biometrics,**10*(1), 101. 10.2307/3001666

[CR27] Coakley, R., & Wihak, T. (2017). Evidence-based psychological interventions for the management of pediatric chronic pain: New directions in research and clinical practice. *Children,**4*(2), 9. 28165415 10.3390/children4020009 PMC5332911

[CR28] Coppola, G., Foschino Barbaro, M. G., Curci, A., Simeone, M., Costantini, A., Goffredo, M., Latrofa, A., Di Liso, D., & Silverman, W. K. (2018). The associations of parents’ and children’s anxiety sensitivity with child anxiety and somatic-hypochondriac symptoms. *Child & Youth Care Forum,**47*(6), 845–861.

[CR29] De Rosnay, M., Cooper, P. J., Tsigaras, N., & Murray, L. (2006). Transmission of social anxiety from mother to infant: An experimental study using a social referencing paradigm. *Behaviour Research and Therapy,**44*(8), 1165–1175. 10.1016/j.brat.2005.09.003 16288978 10.1016/j.brat.2005.09.003

[CR30] Deacon, B. J., Valentiner, D. P., Gutierrez, P. M., & Blacker, D. (2002). The anxiety sensitivity index for children: Factor structure and relation to panic symptoms in an adolescent sample. *Behaviour Research and Therapy,**40*(7), 839–852. 10.1016/s0005-7967(01)00076-6 12074377 10.1016/s0005-7967(01)00076-6

[CR31] DeCoster, J. (2009). Meta-analysis notes. Retrieved from http://www.stat-help.com/notes.html.

[CR32] Dehon, C., Weems, C. F., Stickle, T. R., Costa, N. M., & Berman, S. L. (2005). A cross-sectional evaluation of the factorial invariance of anxiety sensitivity in adolescents and young adults. *Behaviour Research and Therapy,**43*(6), 799–810. 10.1016/j.brat.2004.06.008 15890170 10.1016/j.brat.2004.06.008

[CR33] Drake, K. L., & Kearney, C. A. (2008). Child anxiety sensitivity and family environment as mediators of the relationship between parent psychopathology, parent anxiety sensitivity, and child anxiety. *Journal of Psychopathology and Behavioral Assessment,**30*(2), 79–86.

[CR34] Dubi, K., Rapee, R. M., Emerton, J. L., & Schniering, C. A. (2008). Maternal modeling and the acquisition of fear and avoidance in toddlers: Influence of stimulus preparedness and child temperament. *Journal of Abnormal Child Psychology,**36*(4), 499–512. 10.1007/s10802-007-9195-3 18080181 10.1007/s10802-007-9195-3

[CR35] Dunne, G., & Askew, C. (2013). Vicarious learning and unlearning of fear in childhood via mother and Stranger models. *Emotion,**13*(5), 974–980. 10.1037/a0032994 23795591 10.1037/a0032994

[CR36] East, A. J., Berman, M. E., & Stoppelbein, L. (2006). Familial association of anxiety sensitivity and psychopathology. *Depression and Anxiety,**24*(4), 264–267. 10.1002/da.20227 17004235

[CR37] Ehlers, A. (1993). Somatic symptoms and panic attacks: A retrospective study of learning experiences. *Behaviour Research and Therapy,**31*(3), 269–278. 8476401 10.1016/0005-7967(93)90025-p

[CR38] Eley, T. C., Gregory, A. M., Clark, D. M., & Ehlers, A. (2007). Feeling anxious: A twin study of panic/somatic ratings, anxiety sensitivity and heartbeat perception in children. *Journal of Child Psychology and Psychiatry,**48*(12), 1184–1191. 18093023 10.1111/j.1469-7610.2007.01838.x

[CR39] Erozkan, A. (2012). Examination of relationship between anxiety sensitivity and parenting styles in adolescents. *Educational Sciences: Theory & Practice,**12*, 52–57.

[CR40] Essau, C. A., Ishikawa, S., & Sasagawa, S. (2011). Early learning experience and adolescent anxiety: A cross-cultural comparison between Japan and England. *Journal of Child and Family Studies,**20*(2), 196–204. 10.1007/s10826-010-9404-5

[CR41] Essau, C. A., Leung, P. W. L., Conradt, J., Cheng, H., & Wong, T. (2008). Anxiety symptoms in Chinese and German adolescents: Their relationship with early learning experiences, perfectionism, and learning motivation. *Depression and Anxiety,**25*(9), 801–810. 10.1002/da.20334 17592617 10.1002/da.20334

[CR42] Essau, C. A., Sasagawa, S., & Ollendick, T. H. (2010). The facets of anxiety sensitivity in adolescents. *Journal of Anxiety Disorders,**24*(1), 23–29. 10.1016/j.janxdis.2009.08.001 19713072 10.1016/j.janxdis.2009.08.001

[CR43] Farris, S. G., DiBello, A. M., Allan, N. P., Hogan, J., Schmidt, N. B., & Zvolensky, M. J. (2015). Evaluation of the anxiety sensitivity index-3 among treatment-seeking smokers. *Psychological Assessment,**27*(3), 1123–1128. 10.1037/pas0000112 25894700 10.1037/pas0000112 PMC4549201

[CR44] Fernández-Valdés, J., Gavino, A., Valderrama-Martos, L., Godoy, A., & Laurent, J. (2017). Psychometric properties of the Spanish version of the anxiety sensitivity index for children. *Psicothema,**3*(29), 402–407. 10.7334/psicothema2017.78 10.7334/psicothema2017.78 28693714

[CR45] Field, A. P. (2006). Is conditioning a useful framework for understanding the development and treatment of phobias? *Clinical Psychology Review,**26*(7), 857–875. 10.1016/j.cpr.2005.05.010 16472895 10.1016/j.cpr.2005.05.010

[CR46] Field, A. P., Argyris, N. G., & Knowles, K. A. (2001). Who’s afraid of the big bad wolf: A prospective paradigm to test Rachman’s indirect pathways in children. *Behaviour Research and Therapy,**39*(11), 1259–1276. 10.1016/s0005-7967(00)00080-2 11686263 10.1016/s0005-7967(00)00080-2

[CR47] Field, A., & Lawson, J. (2003). Fear information and the development of fears during childhood: Effects on implicit fear responses and behavioural avoidance. *Behaviour Research and Therapy,**41*(11), 1277–1293. 10.1016/s0005-7967(03)00034-2 14527528 10.1016/s0005-7967(03)00034-2

[CR48] Field, A. P., & Purkis, H. M. (2011). The role of learning in the etiology of child and adolescent fear and anxiety. In W. K. Silverman & A. P. Field (Eds.), *Anxiety disorders in children and adolescents: Research, assessment and intervention* (2nd ed., pp. 227–256). Cambridge University Press.

[CR49] Fisak, B., & Grills-Taquechel, A. E. (2007). Parental modeling, reinforcement, and information transfer: Risk factors in the development of child anxiety? *Clinical Child and Family Psychology Review,**10*(3), 213–231. 10.1007/s10567-007-0020-x 17487582 10.1007/s10567-007-0020-x

[CR50] Field, A. P., & Storksen-Coulson, H. (2007). The interaction of pathways to fear in childhood anxiety: A preliminary study. *Behaviour Research and Therapy,**45*(12), 3051–3059. 10.1016/j.brat.2007.09.001 17935694 10.1016/j.brat.2007.09.001

[CR51] Francis, S. E., & Noël, V. (2010). Parental contributions to child anxiety sensitivity: A review and recommendations for future directions. *Child Psychiatry & Human Development,**41*(6), 595–613. 10.1007/s10578-010-0190-5 20499157 10.1007/s10578-010-0190-5

[CR52] Fullana, M. A., Servera, M., Weems, C. F., Tortella-Feliu, M., & Caseras, X. (2003). Psychometric properties of the childhood anxiety sensitivity index in a sample of Catalan school children. *Anxiety, Stress & Coping,**16*(1), 99–107. 10.1080/1061580021000009647

[CR53] Gardner, C., & Epkins, C. C. (2012). Girls’ rumination and anxiety sensitivity: Are they related after controlling for girl, maternal, and parenting factors? *Child & Youth Care Forum,**41*(6), 561–578. 10.1007/s10566-012-9188-4

[CR54] Gerull, F. C., & Rapee, R. M. (2002). Mother knows best: Effects of maternal modelling on the acquisition of fear and avoidance behaviour in toddlers. *Behaviour Research and Therapy,**40*(3), 279–287. 10.1016/s0005-7967(01)00013-4 11863238 10.1016/s0005-7967(01)00013-4

[CR55] Ghisi, M., Bottesi, G., Altoè, G., Razzetti, E., Melli, G., & Sica, C. (2016). Factor structure and psychometric properties of the anxiety sensitivity index-3 in an Italian community sample. *Frontiers in Psychology*. 10.3389/fpsyg.2016.00160 26909057 10.3389/fpsyg.2016.00160 PMC4754426

[CR56] Ginsburg, G. S., & Drake, K. L. (2002). Anxiety sensitivity and panic attack symptomatology among low-income African-American adolescents. *Journal of Anxiety Disorders,**16*(1), 83–96. 12171215 10.1016/s0887-6185(01)00092-5

[CR57] Ginsburg, G. S., Drake, K. L., Tein, J.-Y., Teetsel, R., & Riddle, M. A. (2015). Preventing onset of anxiety disorders in offspring of anxious parents: A randomized controlled trial of a family-based intervention. *American Journal of Psychiatry,**172*(12), 1207–1214. 10.1176/appi.ajp.2015.14091178 26404420 10.1176/appi.ajp.2015.14091178 PMC6013063

[CR58] Ginsburg, G. S., Tein, J.-Y., & Riddle, M. A. (2020). Preventing the onset of anxiety disorders in offspring of anxious parents: A six-year follow-up. *Child Psychiatry & Human Development,**52*(4), 751–760. 10.1007/s10578-020-01080-8 33070244 10.1007/s10578-020-01080-8 PMC8285043

[CR59] Graham, R. A., & Weems, C. F. (2014). Identifying moderators of the link between parent and child anxiety sensitivity: The roles of gender, Positive Parenting, and corporal punishment. *Journal of Abnormal Child Psychology,**43*(5), 885–893. 10.1007/s10802-014-9945-y PMC4393333 25301177

[CR60] Gray, C. M., Carter, R., & Silverman, W. K. (2010). Anxiety symptoms in African American children: Relations with ethnic pride, anxiety sensitivity, and parenting. *Journal of Child and Family Studies,**20*(2), 205–213. 10.1007/s10826-010-9422-3

[CR61] Hale, L. R., & Calamari, J. E. (2006). Panic symptoms and disorder in youth: What role does anxiety sensitivity play? In C. M. Velotis (Ed.), *New developments in anxiety disorders research* (pp. 131–162). Nova Biomedical Books.

[CR62] Harrer, M., Cuijpers, P., Furukawa, T., & Ebert, D. (2021). *Doing meta-analysis with R: A hands-on guide* (1st ed.). Chapman and Hall. 10.1201/9781003107347

[CR63] Hayward, C., Killen, J. D., Kraemer, H. C., & Taylor, C. B. (2000). Predictors of panic attacks in adolescents. *Journal of the American Academy of Child & Adolescent Psychiatry,**39*(2), 207–214. 10.1097/00004583-200002000-00021 10673832 10.1097/00004583-200002000-00021

[CR64] Hedges, L. V., & Olkin, I. (1985). *Statistical methods for meta-analysis*. Elsevier Science.

[CR65] Hedges, L. V., & Vevea, J. L. (1998). Fixed- and random-effects models in meta-analysis. *Psychological Methods,**3*(4), 486–504. 10.1037/1082-989x.3.4.486

[CR66] Hettema, J. M., Bourdon, J. L., Sawyers, C., Verhulst, B., Brotman, M. A., Leibenluft, E., Pine, D. S., & Roberson-Nay, R. (2020). Genetic and environmental risk structure of internalizing psychopathology in Youth. *Depression and Anxiety,**37*(6), 540–548. 10.1002/da.23024 32369878 10.1002/da.23024 PMC7656112

[CR67] Higgins, J. P. (2003). Measuring inconsistency in meta-analyses. *BMJ,**327*(7414), 557–560. 10.1136/bmj.327.7414.557 12958120 10.1136/bmj.327.7414.557 PMC192859

[CR68] Higgins, J. P. T., & Thompson, S. G. (2002). Quantifying heterogeneity in a meta-analysis. *Statistics in Medicine,**21*(11), 1539–1558. 10.1002/sim.1186 12111919 10.1002/sim.1186

[CR69] Hietanen, J. K., Glerean, E., Hari, R., & Nummenmaa, L. (2016). Bodily maps of emotions across child development. *Developmental Science,**19*(6), 1111–1118. 26898716 10.1111/desc.12389

[CR70] Hovenkamp-Hermelink, J. H., van der Veen, D. C., Oude Voshaar, R. C., Batelaan, N. M., Penninx, B. W. J. H., Jeronimus, B. F., Schoevers, R. A., & Riese, H. (2019). Anxiety sensitivity, its stability and longitudinal association with severity of anxiety symptoms. *Scientific Reports*. 10.1038/s41598-019-39931-7 30867472 10.1038/s41598-019-39931-7 PMC6416311

[CR71] Ho, S. M. Y., Dai, D. W., Mak, C., & Liu, K. W. (2018). Cognitive factors associated with depression and anxiety in adolescents: A two-year longitudinal study. *International Journal of Clinical and Health Psychology,**18*(3), 227–234. 10.1016/j.ijchp.2018.04.001 30487928 10.1016/j.ijchp.2018.04.001 PMC6224862

[CR72] Holly, L. E., & Pina, A. A. (2015). Variations in the influence of parental socialization of anxiety among clinic referred children. *Child Psychiatry & Human Development,**46*(3), 474–484. 25159312 10.1007/s10578-014-0487-x

[CR73] Joiner, T. E., Jr., Schmidt, N. B., Schmidt, K. L., Laurent, J., Catanzaro, S. J., Perez, M., & Pettit, J. W. (2002). Anxiety sensitivity as a specific and unique marker of anxious symptoms in youth psychiatric inpatients. *Journal of Abnormal Child Psychology,**30*(2), 167–175. 12008656 10.1023/a:1014757300294

[CR74] Kearney, C. A., Albano, A. M., Eisen, A. R., Allan, W. D., & Barlow, D. H. (1997). The phenomenology of panic disorder in youngsters: An empirical study of a clinical sample. *Journal of Anxiety Disorders,**11*(1), 49–62. 10.1016/s0887-6185(96)00034-5 9131881 10.1016/s0887-6185(96)00034-5

[CR75] King, N. J., Eleonora, G., & Ollendick, T. H. (1998). Etiology of childhood phobias: Current status of Rachman’s three pathways theory. *Behaviour Research and Therapy,**36*(3), 297–309. 9642849 10.1016/s0005-7967(98)00015-1

[CR76] Kirkby, K. C., Menzies, R. G., Daniels, B. A., & Smith, K. L. (1995). Aetiology of spider phobia: Classificatory differences between two origins instruments. *Behaviour Research and Therapy,**33*(8), 955–958. 10.1016/0005-7967(95)00010-u 7487855 10.1016/0005-7967(95)00010-u

[CR78] Knapp, A. A., Blumenthal, H., Mischel, E. R., Badour, C. L., & Leen-Feldner, E. W. (2016). Anxiety sensitivity and its factors in relation to generalized anxiety disorder among adolescents. *Journal of Abnormal Child Psychology,**44*(2), 233–244. 10.1007/s10802-015-9991-0 25724327 10.1007/s10802-015-9991-0

[CR77] Knapp, A. A., Frala, J., Blumenthal, H., Badour, C. L., & Leen-Feldner, E. W. (2013). Anxiety sensitivity and childhood learning experiences: Impacts on panic symptoms among adolescents. *Cognitive Therapy and Research,**37*(6), 1151–1159.

[CR79] Kramer, L., & Francis, S. (2025). The relationships between adolescent anxiety sensitivity, parent emotional availability, and gender in the context of adolescent anxiety. *Journal of Psychopathology and Behavioral Assessment*. 10.1007/s10862-025-10197-w

[CR80] Lambert, S. F., Cooley, M. R., Campbell, K. D., Benoit, M. Z., & Stansbury, R. (2004). Assessing anxiety sensitivity in inner-city African American children: Psychometric properties of the childhood anxiety sensitivity index. *Journal of Clinical Child & Adolescent Psychology,**33*(2), 248–259. 10.1207/s15374424jccp3302_5 15136188 10.1207/s15374424jccp3302_5

[CR81] Lau, J. J., Calamari, J. E., & Waraczynski, M. (1996). Panic attack symptomatology and anxiety sensitivity in adolescents. *Journal of Anxiety Disorders,**10*(5), 355–364.

[CR82] Lim, Y.-J., & Kim, J.-H. (2012). Korean anxiety sensitivity index-3: Its factor structure, reliability, and validity in non-clinical samples. *Psychiatry Investigation,**9*(1), 45. 10.4306/pi.2012.9.1.45 22396684 10.4306/pi.2012.9.1.45 PMC3285740

[CR83] Lebowitz, E. R., Leckman, J. F., Silverman, W. K., & Feldman, R. (2016). Cross-generational influences on childhood anxiety disorders: Pathways and mechanisms. *Journal of Neural Transmission,**123*(9), 1053–1067. 10.1007/s00702-016-1565-y 27145763 10.1007/s00702-016-1565-y PMC5007197

[CR84] Leen-Feldner, E. W., Blumenthal, H., Babson, K., Bunaciu, L., & Feldner, M. T. (2008). Parenting-related childhood learning history and panic vulnerability: A test using a laboratory-based biological challenge procedure. *Behaviour Research and Therapy,**46*(9), 1009–1016. 18675403 10.1016/j.brat.2008.06.002

[CR85] Leen-Feldner, E. W., Reardon, L. E., McKee, L. G., Feldner, M. T., Babson, K. A., & Zvolensky, M. J. (2006). The interactive role of anxiety sensitivity and pubertal status in predicting anxious responding to bodily sensations among adolescents. *Journal of Abnormal Child Psychology,**34*(6), 797–810. 10.1007/s10802-006-9079-y 10.1007/s10802-006-9079-y 17115272

[CR86] Levy, R. L., Langer, S. L., Walker, L. S., Romano, J. M., Christie, D. L., Youssef, N., DuPen, M. M., Ballard, S. A., Labus, J., Welsh, E., Feld, L. D., & Whitehead, W. E. (2013). Twelve-month follow-up of cognitive behavioral therapy for children with functional abdominal pain. *JAMA Pediatrics,**167*(2), 178. 10.1001/2013.jamapediatrics.282 23277304 10.1001/2013.jamapediatrics.282 PMC4167735

[CR87] Levy, R. L., Langer, S. L., Walker, L. S., Romano, J. M., Christie, D. L., Youssef, N., DuPen, M. M., Feld, A. D., Ballard, S. A., Welsh, E. M., Jeffery, R. W., Young, M., Coffey, M. J., & Whitehead, W. E. (2010). Cognitive-behavioral therapy for children with functional abdominal pain and their parents decreases pain and other symptoms. *American Journal of Gastroenterology,**105*(4), 946–956. 20216531 10.1038/ajg.2010.106 PMC2887246

[CR88] Marin, M.-F., Bilodeau-Houle, A., Morand-Beaulieu, S., Brouillard, A., Herringa, R. J., & Milad, M. R. (2020). Vicarious conditioned fear acquisition and extinction in child–parent dyads. *Scientific Reports*. 10.1038/s41598-020-74170-1 33051522 10.1038/s41598-020-74170-1 PMC7555483

[CR89] Marin, C. E., Rey, Y., Nichols-Lopez, K., & Silverman, W. K. (2008). The relations between anxiety sensitivity and anxiety control in the prediction of anxiety symptoms among children and adolescents. *Behavioural and Cognitive Psychotherapy,**36*(4), 391–402. 10.1017/s1352465808004475

[CR90] Mattis, S. G., & Ollendick, T. H. (1997). Children’s cognitive responses to the somatic symptoms of panic. *Journal of Abnormal Child Psychology,**25*(1), 47–57. 9093899 10.1023/a:1025707424347

[CR91] McLaughlin, E. N., Stewart, S. H., & Taylor, S. (2007). Childhood anxiety sensitivity index factors predict unique variance in DSM-IV anxiety disorder symptoms. *Cognitive Behaviour Therapy,**36*(4), 210–219. 10.1080/16506070701499988 18049946 10.1080/16506070701499988

[CR92] McLaughlin, K. A., & Hatzenbuehler, M. L. (2009). Stressful life events, anxiety sensitivity, and internalizing symptoms in adolescents. *Journal of Abnormal Psychology,**118*(3), 659–669. 10.1037/a0016499 19685962 10.1037/a0016499 PMC2881589

[CR93] McNally, R. J. (2002). Anxiety sensitivity and panic disorder. *Biological Psychiatry,**52*(10), 938–946. 10.1016/s0006-3223(02)01475-0 12437935 10.1016/s0006-3223(02)01475-0

[CR94] McGinn, L. K., Nooner, K. B., Cohen, J., & Leaberry, K. D. (2015). The role of early experience and cognitive vulnerability: Presenting a unified model of the etiology of panic. *Cognitive Therapy and Research,**39*(4), 508–519.

[CR95] McHugh, M. L. (2012). Interrater reliability: The kappa statistic. *Biochemia Medica,**22*, 276–282. 23092060 PMC3900052

[CR96] Methley, A. M., Campbell, S., Chew-Graham, C., McNally, R., & Cheraghi-Sohi, S. (2014). PICO, PICOS and SPIDER: A comparison study of specificity and sensitivity in three search tools for qualitative systematic reviews. *BMC Health Services Research,**14*(1), 1–10. 25413154 10.1186/s12913-014-0579-0 PMC4310146

[CR97] Mineka, S., & Cook, M. (1993). Mechanisms involved in the observational conditioning of fear. *Journal of Experimental Psychology: General,**122*(1), 23–38. 10.1037/0096-3445.122.1.23 8440976 10.1037//0096-3445.122.1.23

[CR98] Mineka, S., & Zinbarg, R. (2006). A contemporary learning theory perspective on the etiology of anxiety disorders: It’s not what you thought it was. *American Psychologist,**61*(1), 10–26. 10.1037/0003-066x.61.1.10 16435973 10.1037/0003-066X.61.1.10

[CR99] Muris, P. (2002). An expanded childhood anxiety sensitivity index: Its factor structure, reliability, and validity in a non-clinical adolescent sample. *Behaviour Research and Therapy,**40*(3), 299–311. 10.1016/s0005-7967(00)00112-1 11863240 10.1016/s0005-7967(00)00112-1

[CR100] Muris, P., & Field, A. P. (2010). The role of verbal threat information in the development of childhood fear. “Beware the jabberwock!” *Clinical Child and Family Psychology Review,**13*(2), 129–150. 10.1007/s10567-010-0064-1 20198423 10.1007/s10567-010-0064-1 PMC2882043

[CR103] Muris, P., Hoeve, I., Meesters, C., & Mayer, B. (2004). Children’s perception and interpretation of anxiety-related physical symptoms. *Journal of Behavior Therapy and Experimental Psychiatry,**35*(3), 233–244. 10.1016/j.jbtep.2004.03.008 15262219 10.1016/j.jbtep.2004.03.008

[CR102] Muris, P., Mayer, B., Freher, N. K., Duncan, S., & van den Hout, A. (2010b). Children’s internal attributions of anxiety-related physical symptoms: Age-related patterns and the role of cognitive development and anxiety sensitivity. *Child Psychiatry & Human Development,**41*(5), 535–548. 10.1007/s10578-010-0186-1 20440551 10.1007/s10578-010-0186-1 PMC2917553

[CR104] Muris, P., & Meesters, C. (2004). Children’s somatization symptoms: Correlations with trait anxiety, anxiety sensitivity, and learning experiences. *Psychological Reports,**94*(3_suppl), 1269–1275. 15362403 10.2466/pr0.94.3c.1269-1275

[CR106] Muris, P., Merckelbach, H., Gadet, B., & Moulaert, V. (2000a). Fears, worries, and scary dreams in 4- to 12-year-old children: Their content, developmental pattern, and origins. *Journal of Clinical Child Psychology,**29*(1), 43–52. 10.1207/s15374424jccp2901_5 10693031 10.1207/S15374424jccp2901_5

[CR107] Muris, P., Merckelbach, H., Mayer, B., & Prins, E. (2000b). How serious are common childhood fears? *Behaviour Research and Therapy,**38*(3), 217–228. 10665156 10.1016/s0005-7967(98)00204-6

[CR105] Muris, P., Merckelbach, H., & Meesters, C. (2001). Learning experiences and anxiety sensitivity in normal adolescents. *Journal of Psychopathology and Behavioral Assessment,**23*(4), 279–283. 10.1023/a:1012783504852

[CR101] Muris, P., van Zwol, L., Huijding, J., & Mayer, B. (2010a). Mom told me scary things about this animal: Parents installing fear beliefs in their children via the verbal information pathway. *Behaviour Research and Therapy,**48*(4), 341–346. 10.1016/j.brat.2009.12.001 20022590 10.1016/j.brat.2009.12.001

[CR108] Muris, P., Vermeer, E., & Horselenberg, R. (2008). Cognitive development and the interpretation of anxiety-related physical symptoms in 4–13-year-old non-clinical children. *Journal of Behavior Therapy and Experimental Psychiatry,**39*(1), 73–86. 17207768 10.1016/j.jbtep.2006.10.014

[CR109] Murray, L., De Rosnay, M., Pearson, J., Bergeron, C., Schofield, E., Royal-Lawson, M., & Cooper, P. J. (2008). Intergenerational transmission of social anxiety: The role of social referencing processes in infancy. *Child Development,**79*(4), 1049–1064. 10.1111/j.1467-8624.2008.01175.x 18717906 10.1111/j.1467-8624.2008.01175.x

[CR110] Naragon-Gainey, K. (2010). Meta-analysis of the relations of anxiety sensitivity to the depressive and anxiety disorders. *Psychological Bulletin,**136*(1), 128–150. 10.1037/a0018055 20063929 10.1037/a0018055

[CR111] Nebbitt, V. E., & Lambert, S. F. (2009). Correlates of anxiety sensitivity among African American adolescents living in urban public housing. *Journal of Community Psychology,**37*(2), 268–280. 10.1002/jcop.20292 10.1002/jcop.22493 33340126

[CR112] Nelles, W. B., & Barlow, D. H. (1988). Do children panic? *Clinical Psychology Review,**8*(4), 359–372. 10.1016/0272-7358(88)90064-5

[CR113] Nimphy, C. A., Mitrou, V., Elzinga, B. M., Van der Does, W., & Aktar, E. (2024). The role of parental verbal threat information in children’s fear acquisition: A systematic review and meta-analysis. *Clinical Child and Family Psychology Review*. 10.1007/s10567-024-00485-4 38789695 10.1007/s10567-024-00485-4 PMC11486780

[CR114] Nimphy, C. A., Venetikidi, M., Elzinga, B., van der Does, W., & Aktar, E. (2023). Parent to offspring fear transmission via modeling in early life: A systematic review and meta-analysis. *Clinical Child and Family Psychology Review,**26*(3), 751–772. 37500947 10.1007/s10567-023-00448-1 PMC10465674

[CR116] Noël, V. A., & Francis, S. E. (2011). A meta-analytic review of the role of child anxiety sensitivity in child anxiety. *Journal of Abnormal Child Psychology,**39*(5), 721–733. 21286803 10.1007/s10802-011-9489-3

[CR117] Olatunji, B. O., & Wolitzky-Taylor, K. B. (2009). Anxiety sensitivity and the anxiety disorders: A meta-analytic review and synthesis. *Psychological Bulletin,**135*(6), 974–999. 10.1037/a0017428 19883144 10.1037/a0017428

[CR118] Ollendick, T. H., & Horsch, L. M. (2007). Fears in clinic-referred children: Relations with child anxiety sensitivity, maternal overcontrol, and maternal phobic anxiety. *Behavior Therapy,**38*(4), 402–411. 18021954 10.1016/j.beth.2006.12.001

[CR119] Ollendick, T. H., & King, N. J. (1991). Origins of childhood fears: An evaluation of Rachman’s theory of fear acquisition. *Behaviour Research and Therapy,**29*(2), 117–123. 2021373 10.1016/0005-7967(91)90039-6

[CR120] Ollendick, T. H., & Muris, P. (2015). The scientific legacy of little Hans and little Albert: Future directions for research on specific phobias in youth. *Journal of Clinical Child & Adolescent Psychology,**44*(4), 689–706. 10.1080/15374416.2015.1020543 25864566 10.1080/15374416.2015.1020543

[CR121] Ollendick, T. H., Yang, B., King, N. J., Dong, Q., & Akande, A. (1996). Fears in American, Australian, Chinese, and Nigerian children and adolescents: A cross-cultural study. *Journal of Child Psychology and Psychiatry,**37*(2), 213–220. 8682901 10.1111/j.1469-7610.1996.tb01393.x

[CR122] Olsson, A., & Phelps, E. A. (2007). Social learning of fear. *Nature Neuroscience,**10*(9), 1095–1102. 17726475 10.1038/nn1968

[CR177] Öst, L.-G., & Hugdahl, K. (1981). Acquisition of phobias and anxiety response patterns in clinical patients. *Behaviour Research and Therapy,**19*(5), 439–447. 10.1016/0005-7967(81)90134-0 7316921 10.1016/0005-7967(81)90134-0

[CR123] Ouzzani, M., Hammady, H., Fedorowicz, Z., & Elmagarmid, A. (2016). Rayyan—a web and mobile app for systematic reviews. *Systematic Reviews,**5*(1), 1–10. 27919275 10.1186/s13643-016-0384-4 PMC5139140

[CR124] Page, M. J., McKenzie, J. E., Bossuyt, P. M., Boutron, I., Hoffmann, T. C., Mulrow, C. D., Shamseer, L., Tetzlaff, J. M., Akl, E. A., Brennan, S. E., Chou, R., Glanville, J., Grimshaw, J. M., Hróbjartsson, A., Lalu, M. M., Li, T., Loder, E. W., Mayo-Wilson, E., McDonald, S., … Moher, D. (2021). The Prisma 2020 statement: An updated guideline for reporting systematic reviews. *BMJ*. 10.1136/bmj.n71 33782057 10.1136/bmj.n71 PMC8005924

[CR126] Percy, R., Creswell, C., Garner, M., O’Brien, D., & Murray, L. (2016). Parents’ verbal communication and childhood anxiety: A systematic review. *Clinical Child and Family Psychology Review,**19*(1), 55–75. 10.1007/s10567-015-0198-2 26613935 10.1007/s10567-015-0198-2

[CR127] Qi, J., Rappaport, L. M., Cecilione, J., Hettema, J. M., & Roberson-Nay, R. (2019). Differential associations of distress tolerance and anxiety sensitivity with adolescent internalizing psychopathology. *Journal of Clinical Child Adolescent Psychology,**50*(1), 97–104. 10.1080/15374416.2019.1602838 31059291 10.1080/15374416.2019.1602838 PMC6832780

[CR128] Rabian, B., Embry, L., & MacIntyre, D. (1999). Behavioral validation of the childhood anxiety sensitivity index in children. *Journal of Clinical Child Psychology,**28*(1), 105–112. 10.1207/s15374424jccp2801_9 10070611 10.1207/s15374424jccp2801_9

[CR129] Rachman, S. J. (1977). The conditioning theory of fear acquisition: A critical examination. *Behaviour Research and Therapy,**15*(5), 375–387. 612338 10.1016/0005-7967(77)90041-9

[CR130] Reiss, S. (1991). Expectancy model of fear, anxiety, and panic. *Clinical Psychology Review,**11*(2), 141–153.

[CR131] Reiss, S., Peterson, R. A., Gursky, D. M., & McNally, R. J. (1986). Anxiety sensitivity, anxiety frequency and the prediction of fearfulness. *Behaviour Research and Therapy,**24*(1), 1–8. 3947307 10.1016/0005-7967(86)90143-9

[CR132] Reiss, S., Silverman, W. K., & Weems, C. F. (2001). Anxiety sensitivity. In M. W. Vasey & M. R. Dadds (Eds.), *The developmental psychopathology of anxiety. *Oxford Academic.

[CR133] Rifkin, L. S., Beard, C., Hsu, K. J., Garner, L., & Björgvinsson, T. (2015). Psychometric properties of the anxiety sensitivity index-3 in an acute and heterogeneous treatment sample. *Journal of Anxiety Disorders,**36*, 99–102. 10.1016/j.janxdis.2015.09.010 26460538 10.1016/j.janxdis.2015.09.010

[CR134] Rodriguez, B. F., Bruce, S. E., Pagano, M. E., Spencer, M. A., & Keller, M. B. (2004). Factor structure and stability of the anxiety sensitivity index in a longitudinal study of anxiety disorder patients. *Behaviour Research and Therapy,**42*(1), 79–91. 10.1016/s0005-7967(03)00074-3 14744525 10.1016/s0005-7967(03)00074-3 PMC3272759

[CR135] Sandín, B., Valiente, R. M., Chorot, P., & Santed Germán, M. A. (2007). ASI-3: Nueva Escala para la evaluación de la sensibilidad a la ansiedad. *Revista De Psicopatología y Psicología Clínica*. 10.5944/rppc.vol.12.num.2.2007.4036

[CR136] Scher, C. D., & Stein, M. B. (2003). Developmental antecedents of anxiety sensitivity. *Journal of Anxiety Disorders,**17*(3), 253–269. 10.1016/s0887-6185(02)00202-5 12727121 10.1016/s0887-6185(02)00202-5

[CR137] Schmidt, N. B., Keough, M. E., Mitchell, M. A., Reynolds, E. K., MacPherson, L., Zvolensky, M. J., & Lejuez, C. W. (2010). Anxiety sensitivity: Prospective prediction of anxiety among early adolescents. *Journal of Anxiety Disorders,**24*(5), 503–508. 10.1016/j.janxdis.2010.03.007 20399075 10.1016/j.janxdis.2010.03.007 PMC2872504

[CR138] Schmidt, N. B., Lerew, D. R., & Jackson, R. J. (1997). The role of anxiety sensitivity in the pathogenesis of panic: Prospective evaluation of spontaneous panic attacks during acute stress. *Journal of Abnormal Psychology,**106*(3), 355–364. 10.1037/0021-843x.106.3.355 9241937 10.1037//0021-843x.106.3.355

[CR139] Schmidt, N. B., Lerew, D. R., & Jackson, R. J. (1999). Prospective evaluation of anxiety sensitivity in the pathogenesis of panic: Replication and extension. *Journal of Abnormal Psychology,**108*(3), 532–537. 10.1037/0021-843x.108.3.532 10466277 10.1037//0021-843x.108.3.532

[CR140] Schmidt, N. B., Zvolensky, M. J., & Maner, J. K. (2006). Anxiety sensitivity: Prospective prediction of panic attacks and axis I pathology. *Journal of Psychiatric Research,**40*(8), 691–699. 10.1016/j.jpsychires.2006.07.009 16956622 10.1016/j.jpsychires.2006.07.009

[CR141] Sherman, J. A., Braun, D. A., & Ehrenreich-May, J. (2019). Anxiety sensitivity treatment for children and adolescents. In *The clinician’s guide to anxiety sensitivity treatment and assessment* (pp. 121–144). Academic Press. 10.1016/b978-0-12-813495-5.00007-3

[CR142] Silverman, W. K., Fleisig, W., Rabian, B., & Peterson, R. A. (1991). Childhood anxiety sensitivity index. *Journal of Clinical Child Psychology,**20*(2), 162–168. 10.1207/s15374424jccp2002_7

[CR143] Silverman, W. K., Goedhart, A. W., Barrett, P., & Turner, C. (2003). The facets of anxiety sensitivity represented in the childhood anxiety sensitivity index: Confirmatory analyses of factor models from past studies. *Journal of Abnormal Psychology,**112*(3), 364–374. 10.1037/0021-843x.112.3.364 12943015 10.1037/0021-843x.112.3.364

[CR144] Skversky-Blocq, Y., Haaker, J., & Shechner, T. (2021). Watch and learn: Vicarious threat learning across human development. *Brain Sciences,**11*(10), 1345. 10.3390/brainsci11101345 34679409 10.3390/brainsci11101345 PMC8533719

[CR145] Spinhoven, P., van Hemert, A. M., & Penninx, B. W. (2017). Experiential avoidance and bordering psychological constructs as predictors of the onset, relapse and maintenance of anxiety disorders: One or many? *Cognitive Therapy and Research,**41*(6), 867–880. 10.1007/s10608-017-9856-7 29104331 10.1007/s10608-017-9856-7 PMC5656711

[CR146] Stassart, C., Dardenne, B., & Etienne, A. (2017). The role of parental anxiety sensitivity and learning experiences in children’s anxiety sensitivity. *British Journal of Developmental Psychology,**35*(3), 359–375. 10.1111/bjdp.12172 28120529 10.1111/bjdp.12172

[CR147] Stassart, C., Dardenne, B., & Etienne, A.-M. (2014). Specificity of gender role orientation, biological sex and trait emotional intelligence in child anxiety sensitivity. *Personality and Individual Differences,**71*, 165–170. 10.1016/j.paid.2014.07.040

[CR148] Stein, M. B., Jang, K. L., & Livesley, W. J. (1999). Heritability of anxiety sensitivity: A twin study. *American Journal of Psychiatry,**156*(2), 246–251. 9989561 10.1176/ajp.156.2.246

[CR149] Stewart, S. H., Taylor, S., & Baker, J. M. (1997). Gender differences in dimensions of anxiety sensitivity. *Journal of Anxiety Disorders,**11*(2), 179–200. 10.1016/s0887-6185(97)00005-4 9168341 10.1016/s0887-6185(97)00005-4

[CR150] Stewart, S. H., Taylor, S., Jang, K. L., Cox, B. J., Watt, M. C., Fedoroff, I. C., & Borger, S. C. (2001). Causal modeling of relations among learning history, anxiety sensitivity, and panic attacks. *Behaviour Research and Therapy,**39*(4), 443–456. 11280342 10.1016/s0005-7967(00)00023-1

[CR151] Talkovsky, A. M., & Norton, P. J. (2014). Anxiety sensitivity across four ethnoracial groups in an undergraduate sample. *Cognitive Behaviour Therapy,**44*(1), 33–43. 10.1080/16506073.2014.953568 25243807 10.1080/16506073.2014.953568

[CR153] Taylor, S., & Cox, B. J. (1998). An expanded anxiety sensitivity index. *Journal of Anxiety Disorders,**12*(5), 463–483. 10.1016/s0887-6185(98)00028-0 9801964 10.1016/s0887-6185(98)00028-0

[CR152] Taylor, S., Jang, K. L., Stein, M. B., & Asmundson, G. J. (2008). A behavioral-genetic analysis of health anxiety: Implications for the cognitive-behavioral model of hypochondriasis. *Journal of Cognitive Psychotherapy,**22*(2), 143–153.

[CR154] Taylor, S., Zvolensky, M. J., Cox, B. J., Deacon, B., Heimberg, R. G., Ledley, D. R., Abramowitz, J. S., Holaway, R. M., Sandin, B., Stewart, S. H., Coles, M., Eng, W., Daly, E. S., Arrindell, W. A., Bouvard, M., & Cardenas, S. J. (2007). Robust dimensions of anxiety sensitivity: Development and initial validation of the anxiety sensitivity index-3. *Psychological Assessment,**19*(2), 176–188. 10.1037/1040-3590.19.2.176 17563199 10.1037/1040-3590.19.2.176

[CR155] Tsao, J. C., Myers, C. D., Craske, M. G., Bursch, B., Kim, S. C., & Zeltzer, L. K. (2005). Parent and child anxiety sensitivity: Relationship in a nonclinical sample. *Journal of Psychopathology and Behavioral Assessment,**27*(4), 259–268.

[CR200] U.S. Department of Health and Human Services. (2021, July). Study Quality Assessment Tools: Quality Assessment Tool for Observational Cohort and Cross-Sectional Studies . National Heart Lung and Blood Institute. https://www.nhlbi.nih.gov/health-topics/study-quality-assessment-tools

[CR156] van Widenfelt, B. M., Siebelink, B. M., Goedhart, A. W., & Treffers, P. D. (2002). The Dutch Childhood Anxiety Sensitivity Index: Psychometric properties and factor structure. *Journal of Clinical Child Adolescent Psychology,**31*(1), 90–100. 10.1207/s15374424jccp3101_11 11845655 10.1207/S15374424JCCP3101_11

[CR157] Varela, R. E., Sanchez-Sosa, J. J., Biggs, B. K., & Luis, T. M. (2009). Parenting strategies and socio-cultural influences in childhood anxiety: Mexican, Latin American descent, and European American families. *Journal of Anxiety Disorders,**23*(5), 609–616. 10.1016/j.janxdis.2009.01.012 19264444 10.1016/j.janxdis.2009.01.012

[CR158] Viechtbauer, W. (2005). Bias and efficiency of meta-analytic variance estimators in the random-effects model. *Journal of Educational and Behavioral Statistics,**30*(3), 261–293. 10.3102/10769986030003261

[CR159] Viechtbauer, W. (2010). Conducting meta-analyses in R with the metaphor package. *Journal of Statistical Software*. 10.18637/jss.v036.i03

[CR160] Vujanovic, A. A., Arrindell, W. A., Bernstein, A., Norton, P. J., & Zvolensky, M. J. (2007). Sixteen-item anxiety sensitivity index. *Assessment,**14*(2), 129–143. 10.1177/1073191106295053 17504886 10.1177/1073191106295053

[CR161] Waszczuk, M. A., Zavos, H. M. S., & Eley, T. C. (2013). Genetic and environmental influences on relationship between anxiety sensitivity and anxiety subscales in children. *Journal of Anxiety Disorders,**27*(5), 475–484. 10.1016/j.janxdis.2013.05.008 23872507 10.1016/j.janxdis.2013.05.008 PMC3878378

[CR162] Walsh, T. M., Stewart, S. H., McLaughlin, E., & Comeau, N. (2004). Gender differences in childhood anxiety sensitivity index (CASI) dimensions. *Journal of Anxiety Disorders,**18*(5), 695–706. 10.1016/s0887-6185(03)00043-4 15275947 10.1016/S0887-6185(03)00043-4

[CR163] Watt, M. C., & Stewart, S. H. (2000). Anxiety sensitivity mediates the relationships between childhood learning experiences and elevated hypochondriacal concerns in young adulthood. *Journal of Psychosomatic Research,**49*(2), 107–118. 11068054 10.1016/s0022-3999(00)00097-0

[CR164] Watt, M. C., O’Connor, R. M., Stewart, S. H., Moon, E. C., & Terry, L. (2008). Specificity of childhood learning experiences in relation to anxiety sensitivity and illness/injury sensitivity: Implications for health anxiety and pain. *Journal of Cognitive Psychotherapy,**22*(2), 128–142.

[CR165] Watt, M. C., Stewart, S. H., & Cox, B. J. (1998). A retrospective study of the learning history origins of anxiety sensitivity. *Behaviour Research and Therapy,**36*(5), 505–525. 9648327 10.1016/s0005-7967(97)10029-8

[CR170] Weems, C. F., Camp, R. D., Neill, E. L., & Scott, B. G. (2021). Developmental differences in child and adolescent reasoning about anxiety sensations. *Cognitive Therapy and Research,**45*(1), 166–178. 10.1007/s10608-020-10182-5 33776171 10.1007/s10608-020-10182-5 PMC7993367

[CR166] Weems, C. F., Hammond-Laurence, K., Silverman, W. K., & Ferguson, C. (1997). The relation between anxiety sensitivity and depression in children and adolescents referred for anxiety. *Behaviour Research and Therapy,**35*(10), 961–966. 9401137 10.1016/s0005-7967(97)00049-1

[CR171] Weems, C. F., Hammond-Laurence, K., Silverman, W. K., & Ginsburg, G. S. (1998). Testing the utility of the anxiety sensitivity construct in children and adolescents referred for anxiety disorders. *Journal of Clinical Child Psychology,**27*(1), 69–77. 10.1207/s15374424jccp2701_8 9561939 10.1207/s15374424jccp2701_8

[CR168] Weems, C. F., Hayward, C., Killen, J., & Taylor, C. B. (2002). A longitudinal investigation of anxiety sensitivity in adolescence. *Journal of Abnormal Psychology,**111*(3), 471–477. 12150423

[CR167] Weems, C. F., Silverman, W. K., & La Greca, A. M. (2000). What do youth referred for anxiety problems worry about? Worry and its relation to anxiety and anxiety disorders in children and adolescents. *Journal of Abnormal Child Psychology,**28*(1), 63–72. 10.1023/a:1005122101885 10772350 10.1023/a:1005122101885

[CR169] Weems, C. F., Taylor, L. K., Marks, A. B., & Varela, R. E. (2010). Anxiety sensitivity in childhood and adolescence: Parent reports and factors that influence associations with child reports. *Cognitive Therapy and Research,**34*(4), 303–315.

[CR172] Wheaton, M. G., Deacon, B. J., McGrath, P. B., Berman, N. C., & Abramowitz, J. S. (2012). Dimensions of anxiety sensitivity in the anxiety disorders: Evaluation of the ASI-3. *Journal of Anxiety Disorders,**26*(3), 401–408. 10.1016/j.janxdis.2012.01.002 22306133 10.1016/j.janxdis.2012.01.002

[CR173] Wolitzky-Taylor, K., Guillot, C. R., Pang, R. D., Kirkpatrick, M. G., Zvolensky, M. J., Buckner, J. D., & Leventhal, A. M. (2015). Examination of anxiety sensitivity and distress tolerance as transdiagnostic mechanisms linking multiple anxiety pathologies to alcohol use problems in adolescents. *Alcoholism: Clinical and Experimental Research,**39*(3), 532–539. 10.1111/acer.12638 25706521 10.1111/acer.12638 PMC4411227

[CR174] Zagustin, T. K. (2013). The role of cognitive behavioral therapy for chronic pain in adolescents. *PM&R,**5*(8), 697–704. 10.1016/j.pmrj.2013.05.009 23953015 10.1016/j.pmrj.2013.05.009

[CR175] Zavos, H. M., Rijsdijk, F. V., & Eley, T. C. (2012). A longitudinal, genetically informative, study of associations between anxiety sensitivity, anxiety and depression. *Behavior Genetics,**42*(4), 592–602. 10.1007/s10519-012-9535-0 22437876 10.1007/s10519-012-9535-0

[CR176] Zvolensky, M. J., Arrindell, W. A., Taylor, S., Bouvard, M., Cox, B. J., Stewart, S. H., Sandin, B., Cardenas, S. J., & Eifert, G. H. (2003). Anxiety sensitivity in six countries. *Behaviour Research and Therapy,**41*(7), 841–859. 10.1016/s0005-7967(02)00187-0 12781249 10.1016/s0005-7967(02)00187-0

